# Influence of External Electric Field on B_6_C_6_N_6_: Focusing on Structure, Electronic Structure, Spectra and Reactivity

**DOI:** 10.3390/molecules31203627

**Published:** 2026-10-12

**Authors:** Xin Chen, Li Yang

**Affiliations:** 1 School of Materials and Chemical Engineering, West Anhui University, Lu’an 237012, China; cx810101@163.com; 2 Training Department, West Anhui University, Lu’an 237012, China

**Keywords:** B_6_C_6_N_6_, external electric fields, UV–Vis spectra, reactivity

## Abstract

Ternary B_6_C_6_N_6_ monocyclic compounds remain purely theoretical, with no experimental synthesis reported, and their behavior under external electric fields (EEFs) is largely unexplored. Taking the *D*_3*h*_-symmetric B_6_C_6_N_6_ isomer as a model, EEF-induced changes in geometry, bonding, electronic structure, excited-state properties, and reactivity were calculated at the ωB97XD/def2-TZVP level combined with TD-DFT (time-dependent density functional theory) and wavefunction analyses. As the EEF strength increases from 0 to 0.029 a.u. (−F_y_ direction), B_6_C_6_N_6_ undergoes a regular *D*_3*h*_→*C*_2*ν*_ symmetry reduction, with the N10–N15 bond at −F_y_ EEF source elongated from 1.28 Å to 1.46 Å, the cavity diameter contracted from 4.065 to 1.201 Å, and the long axis length expanded from 7.631 Å to 9.843 Å, and charge displacement reaches 0.88 e at −0.029 a.u. The HOMO–LUMO gap narrows from 7.81 eV to 5.03 eV, and the lowest-energy absorption band red-shifts from 316.3 nm to 679.8 nm, with a transition from local to charge-transfer excitation. OWDD (orbital-weighted dual descriptor) and LEAE (local electron attachment energy) analyses imply that −F_y_ EEF spatially reshapes the reactive sites, with the LEAE value at preferred nucleophilic sites decreasing to −7.07 eV at −0.029 a.u. These results provide a theoretical basis for understanding and potentially regulating B_6_C_6_N_6_ isomers via EEFs.

## 1. Introduction

The ternary B_x_C_y_N_z_ compounds are expected to integrate the properties of BN, carbon nitride (C_3_N_4_), B_4_C, and graphene [1]. The ratio of x, y and z in B_x_C_y_N_z_ compounds will regulate their properties [1]. Up to now, synthesis, properties and applications of B_x_C_y_N_z_ compounds have mainly focused on 0D (0-dimensional), 1D, 2D, 3D cubic and hexagonal nanomaterials (c-BCN and h-BCN) [2,3,4,5]. Various methods (chemical vapor deposition, physical vapor deposition, pyrolysis, molten salt method) have been developed for synthesizing B_x_C_y_N_z_ compounds, and their applications in many fields have been comprehensively studied. The application fields include photocatalysts and adsorbents, electrocatalysts, supercapacitors, electrochemical sensing, lithium-ion batteries, electromagnetic wave absorption materials, photodetectors, field-effect transistors and so on [1,2,3,4,5,6,7,8,9,10,11].

By contrast, far fewer studies have been reported on B_x_C_y_N_z_ monocyclic compounds, especially on their syntheses. The studies of the B_6_C_6_N_6_ 18-membered ring are currently purely theoretical, and no experimental synthesis has been reported. However, the synthesis of C_18_ [12,13] and BCN heterocyclic compounds [14,15] can provide inspiration for its preparation. B_6_C_6_N_6_ possesses a large number of isomers; present theoretical investigations on B_6_C_6_N_6_ are also limited. The open-shell singlet *C*_6*h*_ B_6_C_6_N_6_ has similar double aromatic character to the C_18_ ring [16], but many other B_6_C_6_N_6_ isomers have no aromaticity [17]. The bridging mode of carbon atoms exerts a significant influence on the electronic delocalization characteristics and aromaticity of these isomers [16,17]. There remain substantial research gaps regarding the synthesis, properties and applications of B_6_C_6_N_6_ isomers.

External electric field (EEF) has been widely used in regulating chemical structure, catalyzing chemical reactions, and controlling stereochemistry [18,19,20]. Little is known about EEF effects on the structure, electronic structure, spectral properties and reactivity of B_6_C_6_N_6_ isomers. Taking the *D*_3*h*_-symmetric B_6_C_6_N_6_ isomer as a model compound, this work studies EEF-induced changes in the above-mentioned properties, so as to offer a theoretical basis for the potential applications of B_6_C_6_N_6_ isomers.

In this work, the upper limit of 0.029 a.u. (1.49 V/Å) was set because further increasing the EEF strength led to SCF (self-consistent field) convergence difficulties, and a similar EEF limit has been used in previous studies on related systems [21].

## 2. Computational Details

Geometry optimizations and frequency calculations were performed at the ωB97XD [22]/def2-TZVP [23] level under different EEF strengths using Gaussian 16 Revision C.02 [24]. EEFs were introduced via the “Field” keyword in Gaussian 16 software. The sign convention of the Gaussian “Field” keyword is opposite to that of many other quantum chemistry packages [25]; “Field = y + N” corresponds to an EEF of N × 10^−4^ a.u., along the −y direction of the standard orientation coordinate system. Accordingly, EEFs of F_y_ = −0.01, −0.02, and −0.029 a.u. were implemented using “Field = y + 100”, “Field = y + 200”, and “Field = y + 290”, respectively. Default convergence criteria and SCF convergence settings were adopted for geometry optimizations. Frequency calculations confirmed the absence of imaginary modes for all optimized structures under all studied EEF strengths.

UV-Vis spectra were simulated via TD-DFT [26,27] calculations at the TD-ωB97XD/def2-TZVP level with Gaussian 16 Revision C.02 [24]. All remaining analyses were performed using Multiwfn 3.8 (dev) [28,29]. Except for color-filled LOL (localized orbital locator)-π maps, color-filled maps of the electron localization function (ELF) and LEAE isosurfaces rendered by VMD (Visual Molecular Dynamics) [30], all other visual maps were generated using Multiwfn 3.8 (dev) [28,29]. The ωB97XD/def2-TZVP theoretical level was selected according to numerous previously reported related studies [16,17]. All molecular thermodynamic quantities were obtained from the Shermo program [31].

## 3. Results and Discussions

### 3.1. Geometry Evolution Under +F_y_ and −F_y_ EEFs

At zero field (0 a.u.), B_6_C_6_N_6_ adopts a *D*_3*h*_ circular conformation with a zero dipole moment. The long-axis length *D* is 7.631 Å, and bond lengths and bond angles are homogeneously distributed over the ring skeleton (Figure S1 and Table S1).

**Figure 1:**
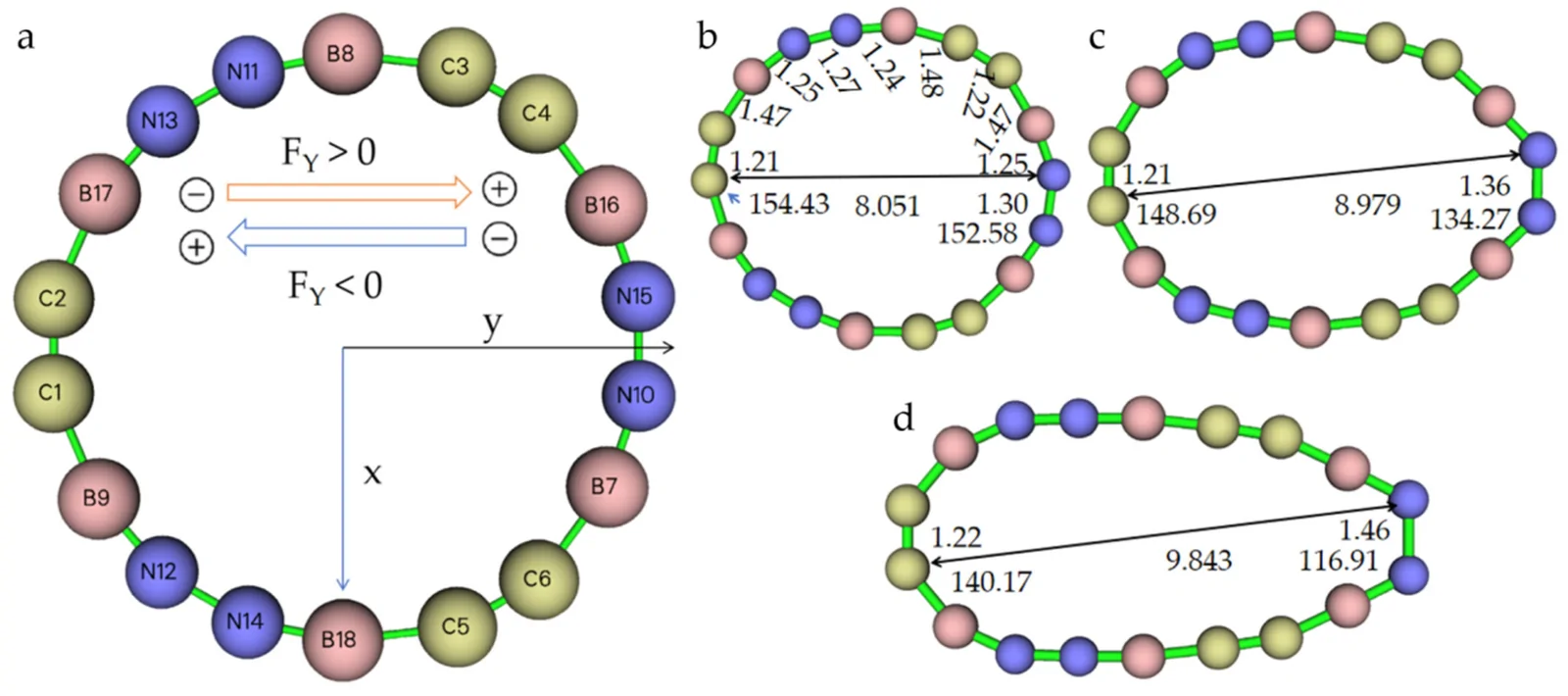
Atomic numbering, molecular coordinate axes and EEF direction (**a**). Optimized structural parameters of B_6_C_6_N_6_ under different −F_y_ EEFs: −0.01 (**b**), −0.02 (**c**), and −0.029 a.u. (**d**). The arrow indicates the molecular size *D* (in Å). Other values are bond lengths (in Å) and bond angles (in °). The purple, orange, and blue spheres represent B, C, and N atoms, respectively.

The same upper limit of 0.029 a.u. was applied to both the +F_y_ and −F_y_ directions (Figure 1). Optimized Cartesian coordinates of B_6_C_6_N_6_ under various EEFs are provided as Supplementary Data S1. With the increase in −F_y_ EEF from 0 to −0.029 a.u., B_6_C_6_N_6_ undergoes symmetry reduction from *D*_3*h*_ to *C*_2*v*_, with the dipole moment rising monotonically from 0 to 45.068 (Table 1). C1–C2 lies along the −F_y_ direction and N10–N15 is at the field origin (Figure 1); their structural response to −F_y_ EEF is anisotropic. The C1–C2 bond length remains nearly constant (1.21→1.22 Å), whereas the N10–N15 bond is strongly elongated (1.28→1.46 Å). The corresponding bond angles also change unevenly. ∠C2–C1–B9 decreases by 16.38° (156.55→140.17°), while ∠N15–N10–B7 decreases by 43.89° (160.80°→116.91°), indicating more severe rearrangement around N10–N15. The molecular longest axis *D* expands from 7.631 to 9.843 Å, whereas the cavity diameter contracts from 4.065 to 1.201 Å, with the most drastic cavity diameter shrinkage occurring between −0.02 and −0.025 a.u. (2.629→1.728 Å). Overall, the −F_y_ EEF selectively weakens N10–N15, elongates the molecular skeleton, compresses the internal cavity, and breaks the original *D*_3*h*_ symmetry, while the C1–C2 bond aligned with the field remains essentially unaffected.

In contrast, the structural response under +F_y_ shows weaker regularity (Figure S1). Within 0.01–0.0275 a.u., B_6_C_6_N_6_ retains *C*_2*v*_ symmetry, with *D* expanding from 7.813 Å to 8.262 Å alongside gradual polarization enhancement. Bond angle distortion becomes less synchronous between the two poles of +F_y_ EEF. ∠C2–C1–B9 shrinks (157.26°, 156.97°, 155.92°, 154.09°, 153.04°, and 140.06° under +F_y_ 0.01, 0.015, 0.02, 0.025, 0.0275, and 0.029 a.u., respectively.), while ∠N15–N10–B7 decreases from 164.77° to 163.37° via a slight increase (Figure S1). When EEF reaches the critical value of +F_y_ 0.029 a.u., abrupt symmetry breaking and deformation occur, yielding *C*_1_ symmetry with a sharply increased *D* of 9.850 Å (Figure S2), and slightly deviating from a planar structure (with molecular planar parameter MPP [32] 0.0027).

In summary, increasing ±F_y_ EEF enhances molecular polarization and triggers bond distortion, angle variation and long-axis expansion. Nevertheless, −F_y_ yields highly regular, *C*_2*v*_-conserved ovalization, whereas +F_y_ exhibits poor deformation regularity and undergoes abrupt symmetry breaking at a critical field. The symmetry-breaking threshold and deformation regularity are strongly field-direction dependent, implying the intrinsic anisotropic structural behavior of B_6_C_6_N_6_ under EEFs. Unless otherwise specified, the subsequent discussion is limited to the EEF in the −F_y_ direction.

### 3.2. Electronic Structure and Bonding Evolution

#### 3.2.1. Bond Order Analyses

Table 2 summarizes four types of bond orders (Mayer (MBO) [33], Wiberg (WBO) [34], Fuzzy (FBO) [35], and Laplacian (LBO) [36]) for the C1–C2 and N10–N15 bonds at the two terminals of B_6_C_6_N_6_ under the −F_y_ EEF. As the −F_y_ EEF increases from 0 to −0.029 a.u., the bond orders of both bonds generally decrease or remain nearly constant, suggesting weaker bonding at both terminals, but to different extents.

These four types of bond orders are not equivalent. MBO, WBO, and FBO are derived from orbital-overlap or electron-partitioning schemes. LBO is defined as a scaled integral of the negative regions of the electron-density Laplacian within the fuzzy overlap space and may be more sensitive to covalent character. This distinction is important for interpreting the results below.

The C1–C2 bond maintains relatively high bond orders (2.25–2.63) under all involved fields, consistent with strong bonding. At −0.029 a.u., its MBO decreases only from 2.55 to 2.25 (≈11.8%), while the LBO changes merely from 2.62 to 2.59 (≈1.1%). This suggests that C1–C2 is relatively insensitive to the −F_y_ EEF, consistent with its nearly unchanged bond length (1.21→1.22 Å).

For the N10–N15 bond, the MBO decreases from 1.03 to 0.79 (≈23.3%), WBO from 1.50 to 1.19 (≈20.7%), FBO from 1.51 to 1.32 (≈12.6%), and LBO from 1.15 to 0.36 (≈68.7%). The LBO decreases much more steeply than the other three bond orders. This may suggest that the −F_y_ EEF affects the covalent character of N10–N15 more strongly, but the LBO should be interpreted together with the other bond order descriptors. The apparent loss of covalent character is consistent with the large elongation of the N10–N15 bond (1.28→1.46 Å) and the decrease in ∠N15–N10–B7 (160.80°→116.91°). Although the LBO value of 0.36 may indicate weakening of this bond, it does not by itself allow a definitive conclusion regarding its bonding situation.

Overall, the four types of bond orders suggest that the −F_y_ EEF may achieve differential regulation of the two terminal bonds, with a more apparent effect on N10–N15 than on C1–C2 in terms of percentage reductions, even though the absolute reductions of MBO, WBO, and FBO are relatively comparable for the two bonds. However, the LBO should be interpreted alongside the other bond order descriptors, and these results should not be over-interpreted as evidence for a specific mechanism of bond weakening.

**Figure 2:**
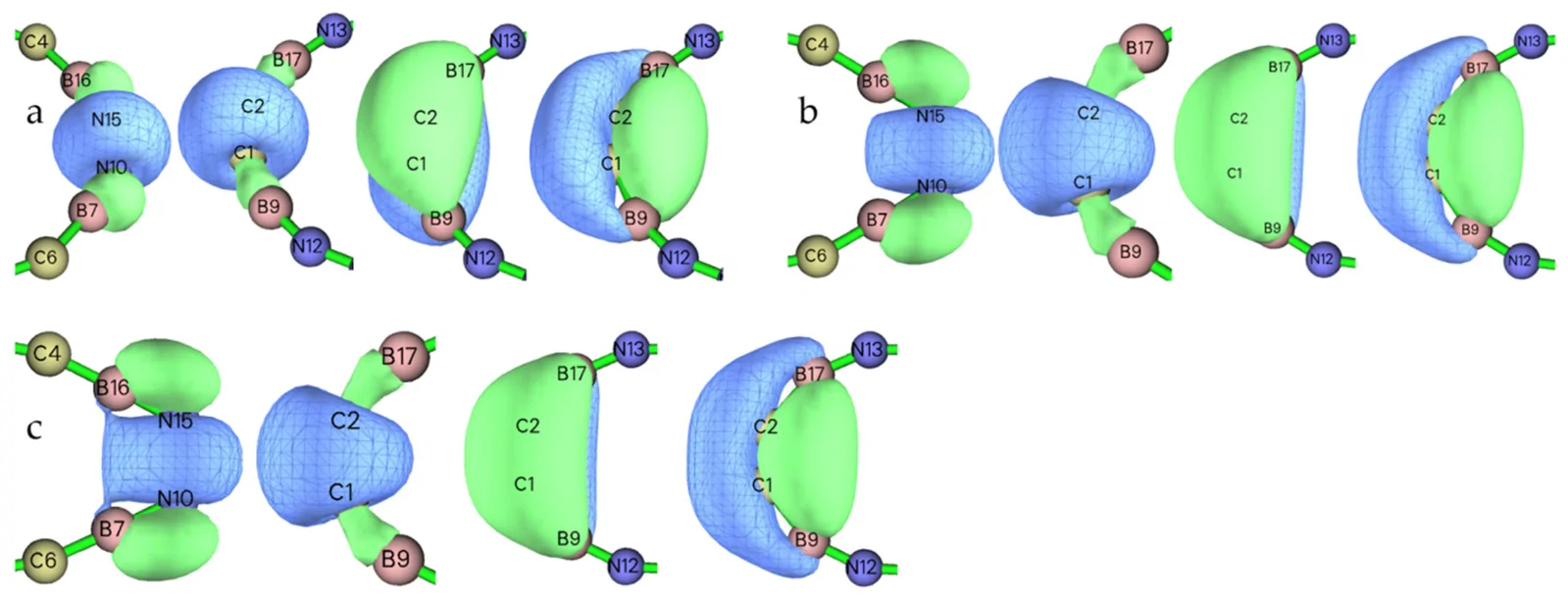
LMO of N10–N15 and C1–C2 bond of B_6_C_6_N_6_ under −0.01 a.u. (**a**), −0.025 a.u. (**b**), and −0.029 a.u. (**c**). Isovalue = 0.03 a.u. The green and blue isosurfaces denote positive and negative values, respectively.

To further characterize the bonding features of C1–C2 and N10–N15, localized molecular orbitals (LMOs) using the Pipek–Mezey method based on Becke population are analyzed at three representative field strengths of −0.01, −0.025, and −0.029 a.u. As can be seen from Figure 2, the C1–C2 bond comprises σ, π_in_, and π_out_ components under all three electric fields, while the N10–N15 bond displays predominantly σ-bond character.

#### 3.2.2. π-Electron Delocalization

The influence of −F_y_ EEF on π-electron delocalization of the B_6_C_6_N_6_ ring was investigated using ELF-π and LOL-π (ELF and LOL [37] are the electron localization functional and localized orbital locator, respectively). LOL-π_in_ and LOL-π_out_ isosurfaces of B_6_C_6_N_6_ under different −F_y_ EEFs are shown in Figure S3 (π_in_ and π_out_ are in-plane and out-of-plane π-electron, respectively).

**Figure 3:**
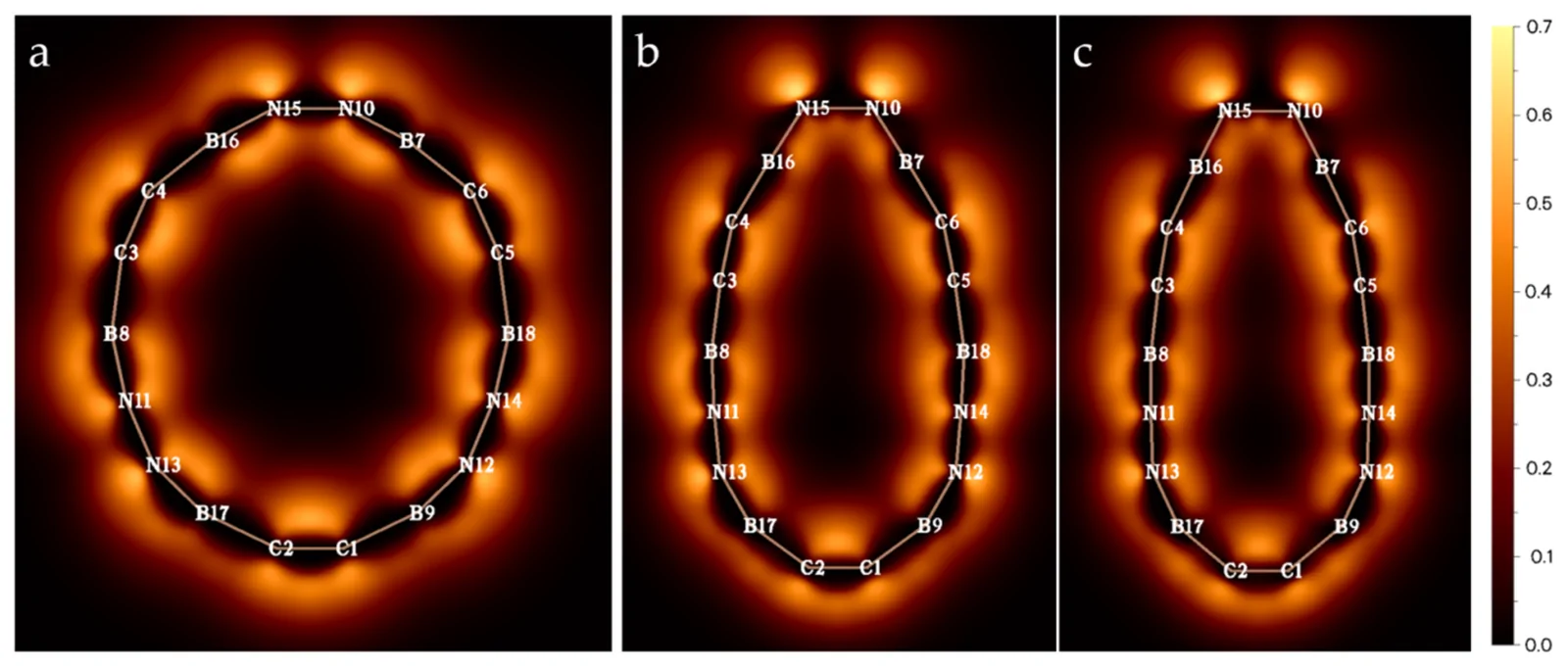
Color-filled LOL-π maps at the B_6_C_6_N_6_ molecular plane under different −F_y_ EEFs ((**a**): −0.01 a.u., (**b**): −0.025 a.u., (**c**): −0.029 a.u.) and color bar.

LOL-π_in_ isosurfaces are present on both the outer and inner sides of the B_6_C_6_N_6_ ring. For clarity, they are termed outer and inner LOL-π_in_ isosurfaces, respectively. As shown in Figure S3, when the isovalue is set to 0.4 a.u., for the LOL-π_in_ isosurfaces, the outer LOL-π_in_ isosurfaces along the N–N bonds remain discontinuous across all applied −F_y_ EEFs with magnitudes from 0.01 to 0.029 a.u. At the weakest field of −0.01 a.u., the inner LOL-π_in_ isosurfaces along the B–C bonds are discontinuous. As the EEF magnitude increases, the inner LOL-π_in_ isosurfaces along most B–C bonds become continuous and spatially extended, whereas the B–C bond on the field-terminal side remains discontinuous. This transition to a continuous isosurface is most pronounced for the B–C bond located on the source side of the −F_y_ EEF. Under weak −F_y_ EEFs, the inner and outer LOL-π_in_ isosurfaces along the B–N bonds exhibit extensive electron-localized regions. With increasing EEF strength, the outer LOL-π_in_ isosurface along the B–N bond on the −F_y_ EEF source gradually becomes discontinuous at −0.02 a.u. For the LOL-π_out_ isosurfaces, the isosurface along the N–N bond on the −F_y_ EEF source stays discontinuous across all field strengths. The LOL-π_out_ isosurfaces of the remaining N–N bonds become continuous as the EEF magnitude increases, indicating enhanced π-electron delocalization. The LOL-π_out_ isosurfaces at other positions remain continuous throughout. Overall, π_out_ electron delocalization at N–N and B–C bonds is weaker than that at the remaining sites of the ring, except for that of the N–N bond on the −F_y_ EEF source. This can also be reflected by color-filled LOL-π maps at 1.2 Å above the B_6_C_6_N_6_ molecular plane (Figure S4) and color-filled LOL-π maps at the B_6_C_6_N_6_ molecular plane (Figure 3) under different −F_y_ EEFs.

Figure S5 presents color-filled ELF maps at the B_6_C_6_N_6_ ring plane. Under zero EEF, high ELF domains are localized along the B–C, C–C and B–N bonds of the cyclic skeleton, indicative of strongly localized bonding electrons. Upon increasing the strength of −F_y_ EEF, the electron is polarized along the −F_y_ EEF direction, and delocalization of electrons within the B_6_C_6_N_6_ ring is enhanced. Meanwhile, electron localization at the two N sites (−F_y_ EEF source) is enhanced (Figure 3 and Figure S5). Collectively, the applied −F_y_ EEF effectively perturbs the real-space electron localization of B_6_C_6_N_6_ and drives electronic redistribution along the field direction.

#### 3.2.3. Electron Density Difference (EDD) and Charge Displacement Curve (CDC)

**Figure 4:**
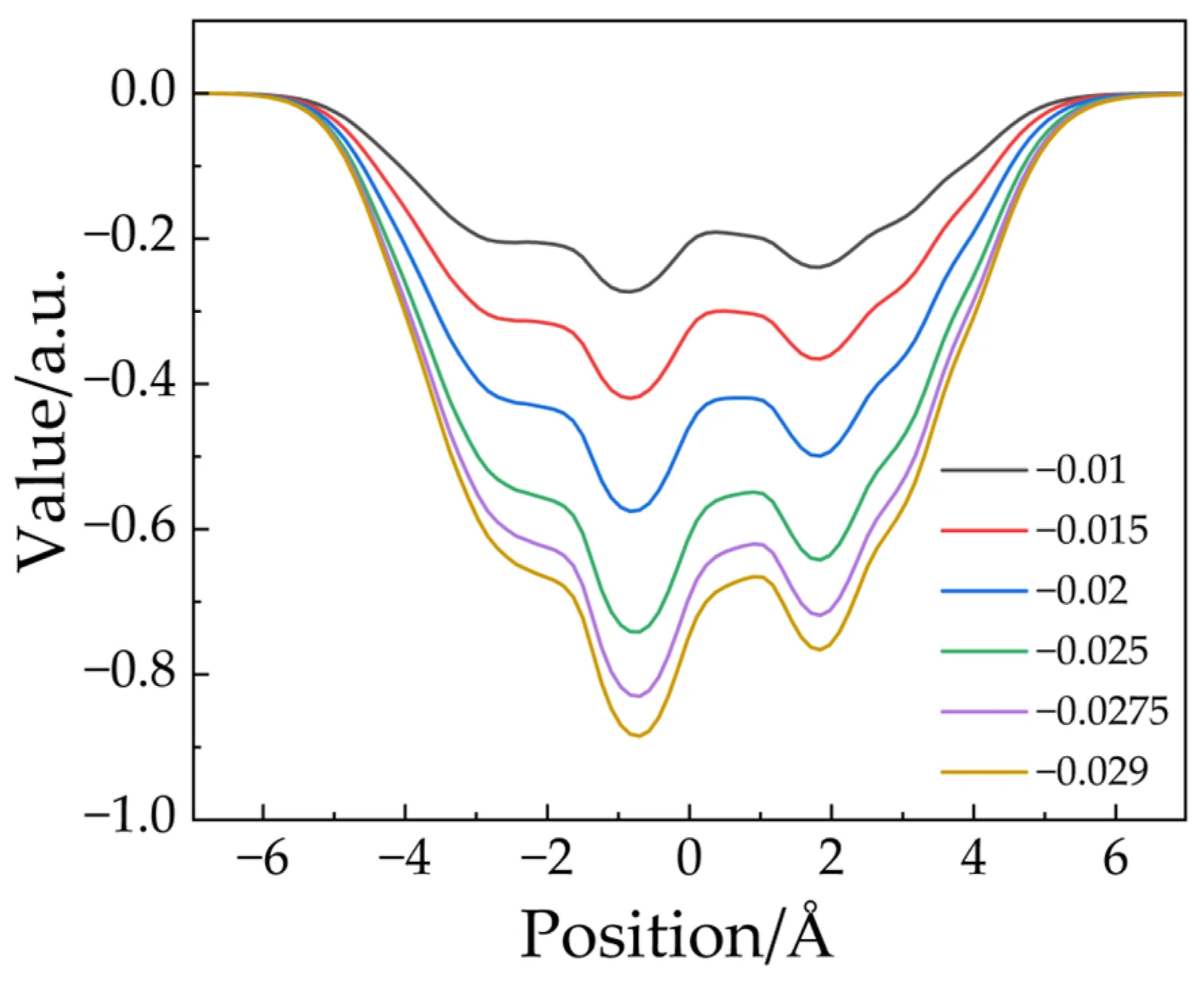
CDC of B_6_C_6_N_6_ under various EEFs in the −F_y_ direction (the values in the figure denote the EEF strength in a.u.).

Plots of EDD (Figure S6) indicate that −F_y_ EEF causes dramatic charge polarization in the B_6_C_6_N_6_ ring, and overall, the electrons markedly move towards the −F_y_ EEF source. The peaks of the local integral curve of B_6_C_6_N_6_ under different −F_y_ EEFs (Figure S7) with values lower and higher than 0 are consistent with the electron gain (green) and loss (blue) isosurfaces in corresponding EDD plots. As seen in Figure 4, the electron transfer amounts within the B_6_C_6_N_6_ ring are 0.27 e (−0.01 a.u.), 0.42 e (−0.015 a.u.), 0.58 e (−0.02 a.u.), 0.74 e (−0.025 a.u.), 0.83 e (−0.0275 a.u.), and 0.88 e (−0.029 a.u.), respectively. The analyses of EDD, CDC and the local integral curve collectively indicate that the −F_y_ EEF induces directional charge migration in B_6_C_6_N_6_, the charge transfer sites are stable, and the transferred charge increases with field strength.

#### 3.2.4. Total Density of States (TDOS) and Partial Density of States (PDOS)

**Figure 5:**
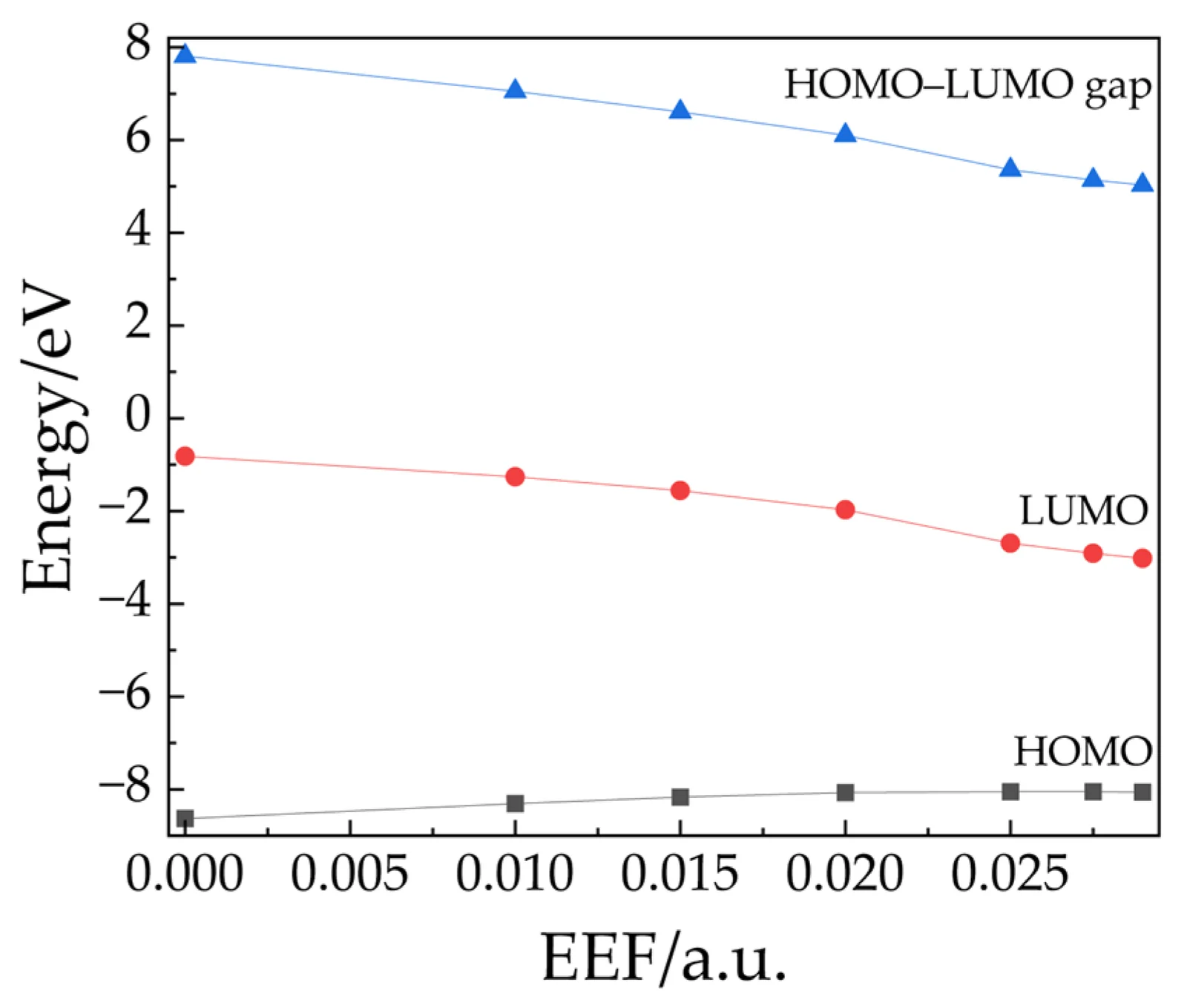
Regulation effect on the energy of HOMO, LUMO and HOMO–LUMO gap of B_6_C_6_N_6_.

At 0 a.u. EEF, B_6_C_6_N_6_ exhibits the lowest HOMO energy of −8.63 eV, the highest LUMO energy of −0.82 eV, and the largest HOMO–LUMO gap of 7.81 eV among all cases (Table S2 and Figure 5). Upon applying −F_y_ EEFs, the LUMO energy steadily decreases and reaches its minimum value of −3.02 eV at −0.029 a.u. The HOMO energy rises moderately, peaks at −8.05 eV under EEFs of −0.025 and −0.0275 a.u., and slightly rebounds afterward. Notably, the LUMO energies remain negative across all investigated EEFs. The HOMO–LUMO gap gradually shrinks with increasing field strength and attains a minimum of 5.03 eV at −0.029 a.u. Figure S8 shows that the HOMO of B_6_C_6_N_6_ is an in-plane π orbital under zero EEF and within the investigated range of −F_y_ EEFs. The LUMO adopts an out-of-plane π orbital character at zero field and low −F_y_ EEF strengths. When the EEF strength reaches −0.02 a.u., the LUMO changes to an in-plane π orbital. The TDOS and PDOS [38,39] maps (Figure S9) show that under zero EEF and all investigated −F_y_ EEFs, the contribution to the HOMO follows the order N > B > C, while the contribution to the LUMO follows B > C > N. Moreover, as the −F_y_ EEF strength increases, the difference in the contributions of N and B to the HOMO decreases, whereas the contribution difference for the LUMO increases.

### 3.3. Excited-State Properties and Optical Responses

**Figure 6:**
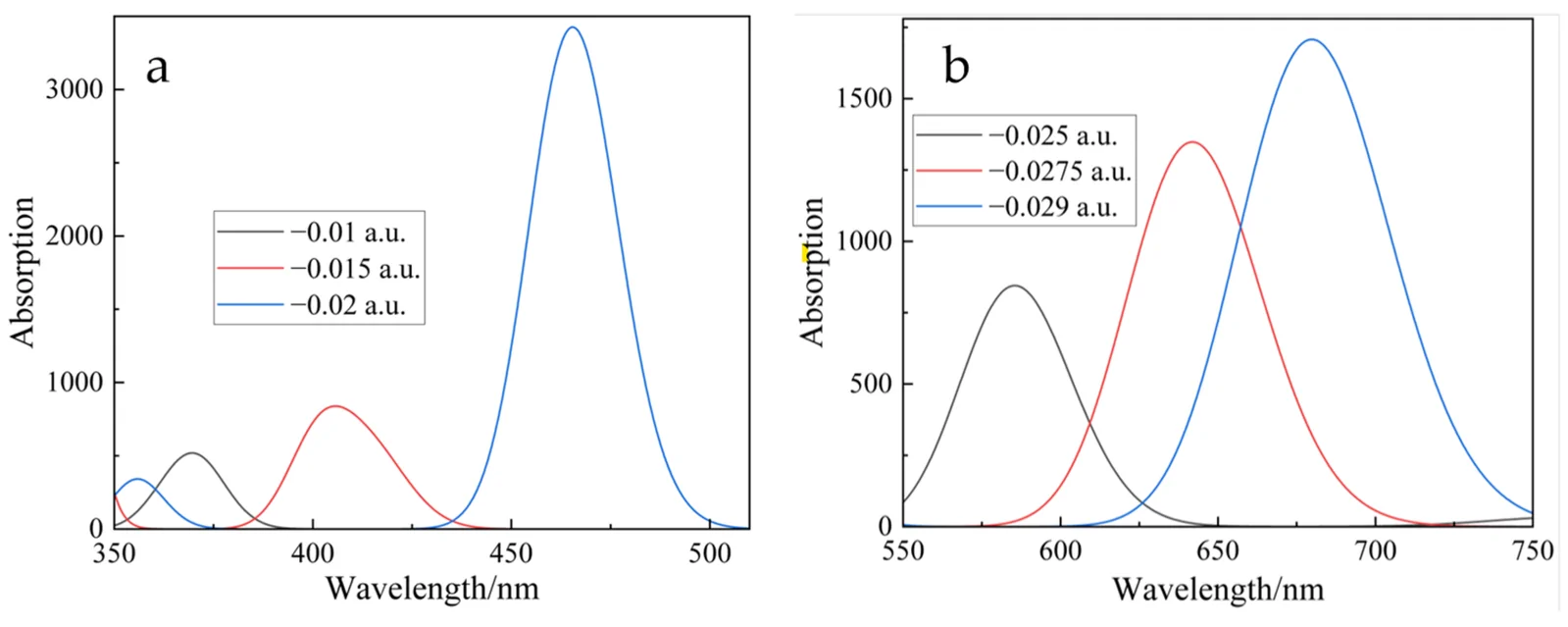
The lowest-energy UV-Vis absorption band of B_6_C_6_N_6_ under different −F_y_ EEFs ((**a**): −0.01 a.u., −0.015 a.u., and −0.02 a.u.; (**b**): −0.025 a.u., −0.0275 a.u., and −0.029 a.u.) with FWHM of 0.3 eV.

The full-range UV–Vis spectra of B_6_C_6_N_6_ in the absence of EEF and under various −F_y_ EEFs (0 to −0.029 a.u.) based on the first 20 excited states, together with the charge transfer spectra (CTS [40,41]) corresponding to the lowest-energy UV–Vis absorption bands (LEAB), are displayed in Figure S10. Figure 6 presents the LEAB of B_6_C_6_N_6_ under different −F_y_ EEFs (−0.01 to −0.029 a.u.). Here, for each −F_y_ EEF, the LEAB is defined as the longest-wavelength peak clearly resolved after Gaussian broadening with the full width at half maximum (FWHM) of 0.3 eV.

As seen in Figure S10 and Table 3, B_6_C_6_N_6_ exhibits a very weak absorption peak at 316.3 nm in the absence of EEF. Among its first 20 excited states, the maximum oscillator strength is only 0.0005, and the oscillator strength (*f*) of all the other excited states is almost 0. When the strength of −F_y_ EEF increases, a strong absorption band appears in the UV region (λ_max_ near 274.4 nm for −0.01 a.u., 249.6 nm for −0.015 a.u., and 299.1 nm for −0.02 a.u., Table S3). Meanwhile, B_6_C_6_N_6_ exhibits its LEAB centered at 371.5 nm (−0.01 a.u.), 416.2 nm (−0.015 a.u.), and 466.1 nm (−0.02 a.u.), respectively. When the −F_y_ EEF exceeds −0.025 a.u., distinct absorption peaks emerge in the visible region (Figure S10a), and the wavelength of LEAB extends to above 550 nm (585.5 nm for −0.025 a.u., 641.9 nm for −0.0275 a.u., and 679.8 nm for −0.029 a.u., Figure 6). The EEF induces splitting and redshift of the absorption peaks of B_6_C_6_N_6_, which is similar to the effect of EEF on its isoelectronic analogue C_18_ [21].

CTS indicate that the contribution ratios for all the LEAB of B_6_C_6_N_6_ under various −F_y_ EEFs are in the same order of electron redistribution in B&N > electron transfer from B&N to C > electron transfer from C to B&N > electron redistribution in C. And the contributions of the former two are dominant; the contributions of electron redistribution in C are almost negligible (Figure S11). As shown in Table 3, the main MO transitions for low-lying excited states of B_6_C_6_N_6_ under various −F_y_ EEF are quite diverse. Hole–electron analyses [42] were performed to characterize the nature of these excited-state transitions.

Judged from Table 4, all the low-lying excited states of B_6_C_6_N_6_ (S4 at −0.01 a.u. and S3 at all other EEFs) have a similar overall mean distribution breadth of electron and hole (with *H* value about 3.35–3.65 Å) under various −F_y_ EEFs. Furthermore, under the same −F_y_ EEF, the hole distribution breadth (HDI, 6.40–9.31) is considerably greater than that of electrons (EDI, 4.89–5.48). With the increase in −F_y_ EEF, the Sr index (threshold [0,1]) gradually changes from 0.757 (−0.01 a.u.) to 0.485 (−0.029 a.u.); the centroid distance (*D*) between holes and electrons increases from 1.503 Å to 3.324 Å. For the *t* value, only the *t* value under −0.01 a.u. is negative (−0.893), and the *t* values are highly positive when −F_y_ EEF is above −0.025 a.u. This indicates that the increase in −F_y_ EEF leads to a decrease in the overlap of hole and electron. Combined with the isosurface plots of hole and electron and C_hole_&C_electron_ (Figure S12), the following conclusions can be drawn. S4 under −0.01 a.u. is LE (local excitation); both of the S3 under −0.015 a.u. and −0.02 a.u. are mixed CT (charge transfer) and LE excitation, with dominant CT character; and all of the S3 under −0.025 a.u., −0.0275 a.u. and −0.029 a.u. are CT excitation.

**Figure 7:**
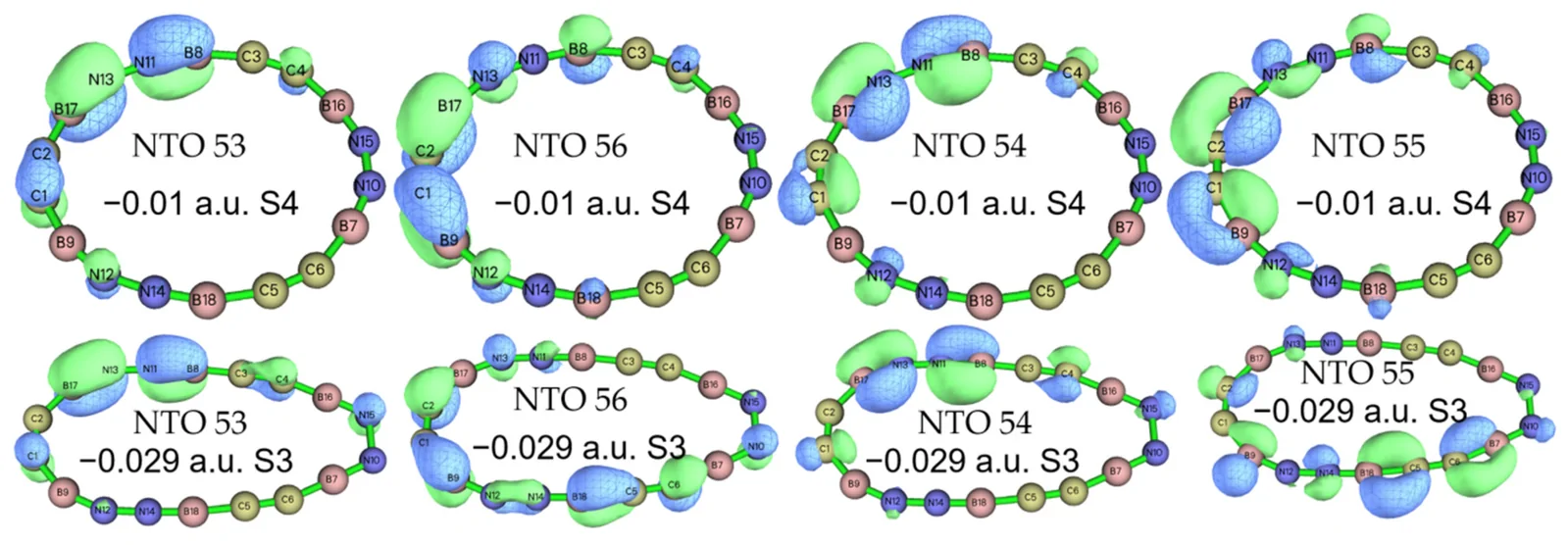
NTO pair plots of S4 under −0.01 a.u. and S3 under −0.029 a.u. The green and blue isosurfaces denote positive and negative values, respectively.

The S0→S4 transition at −0.01 a.u. and the S0→S3 transition at −0.029 a.u. are dominated by natural transition orbitals (NTO [43,44]) 53→NTO 56 and NTO 54→NTO 55, with the corresponding contribution proportions of 39.6% and 45.5%, 13.2% and 84.8%, respectively. NTO analysis (Figure 7) demonstrates that both transitions involve π_out_→π_out_* (NTO 53→NTO 56) and π_in_→π_in_* (NTO 54→NTO 55) features. The two NTO transitions of S4 at −0.01 a.u. are local excitations, while those of S3 at −0.029 a.u. belong to CT excitation. This conclusion is consistent with the results of hole–electron analyses.

### 3.4. Reactivity

**Figure 8:**
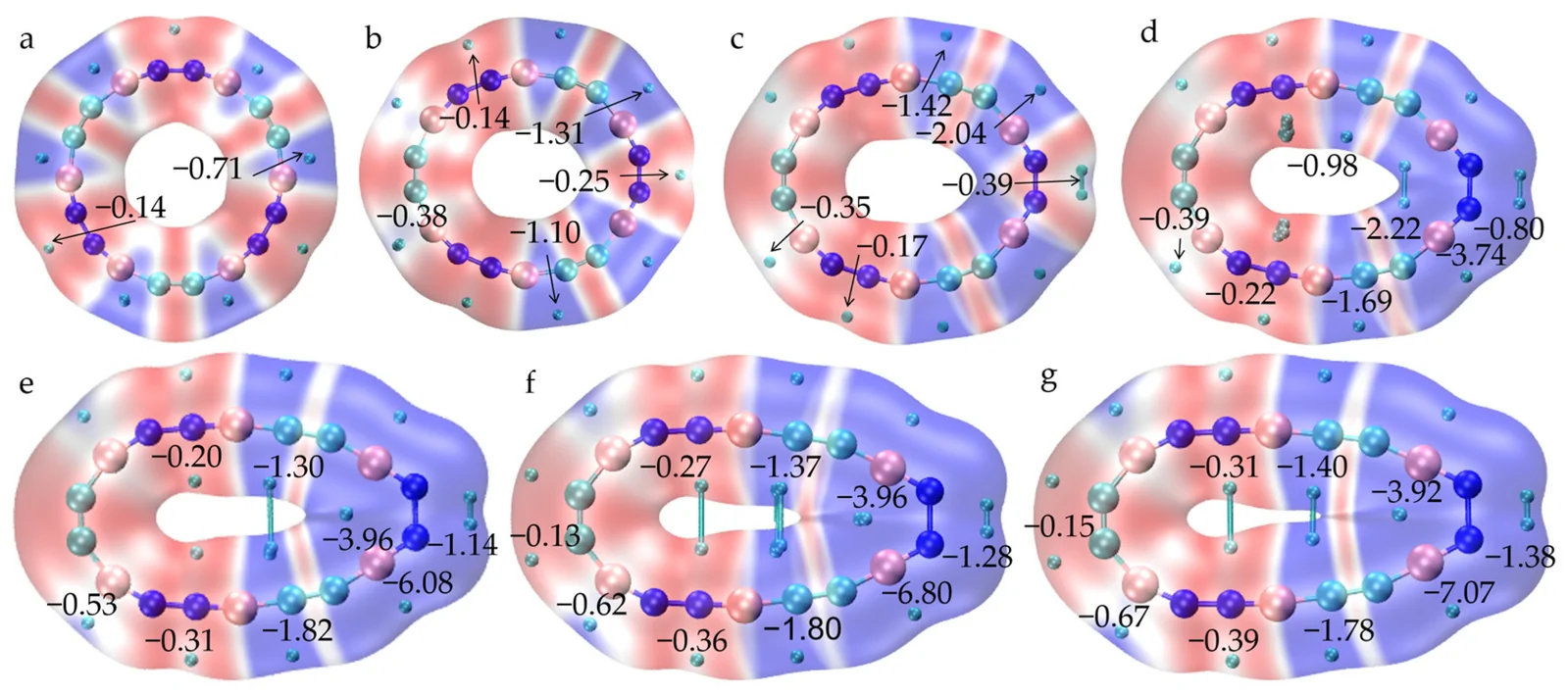
LEAE of B_6_C_6_N_6_ under different EEFs ((**a**) 0; (**b**–**g**) −0.01, −0.015, −0.02, −0.025, −0.0275, and −0.029 a.u.) on the 0.001 a.u. electron density isosurface. All values are in eV.

Isosurface plots of the orbital-weighted dual descriptor (OWDD [45], Figure S13) and local electron attachment energy (LEAE [46], Figure 8) of B_6_C_6_N_6_ under various −F_y_ EEFs are used here as electronic descriptors to predict favorable sites for electrophilic and nucleophilic attack. As shown in Figure S13, under a weak −F_y_ EEF (−0.01 a.u.), the OWDD values at all B–N bonds are negative, suggesting susceptibility to electrophilic attack. In contrast, the OWDD values at all B–C bonds are positive, which may favor nucleophilic attack. As the strength of the −Fy EEF increases (EEF > 0.02 a.u.), the OWDD becomes negative for the B–N and C–C sites on the side towards which the −F_y_ EEF points, while positive OWDD values appear at the B–C and N–N sites on the origin side of the −F_y_ EEF. Moreover, the positive OWDD region at the B–C bonds on the origin side of the −F_y_ EEF expands and extends into the interior of the ring.

Under all the involved −F_y_ EEF strengths, the regions with the most negative LEAE values are located at the origin side of the −F_y_ EEF and on the outer side of the B_6_C_6_N_6_ ring along the B–C bonds closer to the boron atom, which are predicted to be the sites most susceptible to nucleophilic attack. The corresponding LEAE values are −1.31 eV (−0.01 a.u.), −2.04 eV (−0.015 a.u.), −3.74 eV (−0.02 a.u.), −6.08 eV (−0.025 a.u.), −6.80 eV (−0.0275 a.u.), and −7.07 eV (−0.029 a.u.), all substantially more negative than the value of −0.71 eV in the absence of EEF. With increasing −F_y_ EEF strength, the local nucleophilic character at these outer sites is greatly enhanced. For −F_y_ EEF strengths greater than 0.02 a.u., new sites with strongly negative LEAE emerge in the upper inner region of the B_6_C_6_N_6_ ring, between boron atoms adjacent to N–N bonds near the EEF source. Under such EEF conditions, favorable sites for nucleophilic attack are predicted on both the inner and outer sides of the B_6_C_6_N_6_ ring, while the outer side remains the preferred region for nucleophilic attack. These two descriptors collectively imply that the −F_y_ EEF can spatially reshape the landscape of electronic susceptibility of B_6_C_6_N_6_.

It should be noted that these descriptors characterize the intrinsic electronic susceptibility of B_6_C_6_N_6_. Whether a reaction actually occurs at these sites will also depend on the kinetics, identity of the reagent, and solvent effects, which are beyond the scope of the present work.

## 4. Conclusions

In this work, the effects of ±F_y_ EEFs on B_6_C_6_N_6_ up to 0.029 a.u. were explored. The +F_y_ EEFs induced irregular structural deformation and abrupt symmetry breaking at 0.029 a.u. Under −F_y_ EEFs, the molecule underwent relatively regular evolution: the long-axis *D* increased from 7.631 Å to 9.843 Å, the N10–N15 bond showed a −68.7% LBO reduction, and the bandgap narrowed from 7.81 to 5.03 eV. At zero field, B_6_C_6_N_6_ displayed a very weak UV-Vis absorption peak at 316.3 nm. With increasing −F_y_ EEF, the lowest-energy absorption band gradually redshifted to 679.8 nm at −0.029 a.u. The most negative LEAE changed from −0.71 eV (zero field) to −7.07 eV (−0.029 a.u.), yielding more favorable sites for nucleophilic attack.

B_6_C_6_N_6_ exhibited anisotropic responses to +F_y_ and −F_y_ EEFs. These results applied only to the gas-phase *D*_3_*_h_* isomer within the tested field range; solvent, temperature and alternative isomers could modify the observed thresholds and selectivity. The present observations offered insights into EEF-mediated modulation of B_6_C_6_N_6_ and related monocyclic B_x_C_y_N_z_ species, and suggest EEFs may help to tune specific bonds and reactive sites in such rings, which may support further investigations on optical response modulation and identification of reactive sites.

## Figures and Tables

**Table 1:** Main Structural Parameters of B_6_C_6_N_6_ under Various −F_y_ EEFs.

EEF	BL_C1–C2_ (Å)	BL_N10–N15_ (Å)	∠C2–C1–B9 (°)	∠N15–N10–B7 (°)	D (Å)	cav (Å)	Dipole Moment	Point Group
0	1.21	1.28	156.55	160.80	7.631	4.065	0	*D*_3*h*_
−0.01	1.21	1.30	154.43	152.58	8.051	3.710	10.569	*C*_2*v*_
−0.015	1.21	1.32	152.29	145.24	8.429	3.314	17.137	*C*_2*v*_
−0.02	1.21	1.36	148.69	134.27	8.979	2.629	25.706	*C*_2*v*_
−0.025	1.22	1.43	143.67	122.40	9.558	1.728	36.443	*C*_2*v*_
−0.0275	1.22	1.45	141.44	118.70	9.749	1.376	41.821	*C*_2*v*_
−0.029	1.22	1.46	140.17	116.91	9.843	1.201	45.068	*C*_2*v*_

Note: The atom numbering is identical to that of Figure 1a. Cav denotes cavity diameter.

**Table 2:** Four types of bond orders (Mayer, Wiberg, Fuzzy and Laplacian) for C1–C2 and N10–N15 of B_6_C_6_N_6_ under different −F_y_ EEFs.

EEF	Bond Order of C1–C2				Bond Order of N10–N15			
	Mayer	Wiberg	Fuzzy	Laplacian	Mayer	Wiberg	Fuzzy	Laplacian
0	2.55	2.49	2.51	2.62	1.03	1.50	1.51	1.15
−0.01	2.48	2.47	2.50	2.63	0.99	1.46	1.48	1.03
−0.015	2.44	2.45	2.48	2.63	0.95	1.41	1.45	0.91
−0.02	2.38	2.42	2.46	2.63	0.88	1.33	1.41	0.72
−0.025	2.31	2.38	2.42	2.61	0.82	1.24	1.35	0.48
−0.0275	2.28	2.35	2.39	2.60	0.80	1.21	1.33	0.40
−0.029	2.25	2.33	2.37	2.59	0.79	1.19	1.32	0.36

Note: The atom numbering is identical to that of Figure 1a. Cav denotes cavity diameter.

**Table 3:** Photophysical data for the low-lying excited states of B_6_C_6_N_6_ without EEF and under different −F_y_ EEF at gas phase.

EEF	State	Energy/eV	λ/nm	*f*	Main MO Transitions
0	6	3.92	316.31	0.0005	H→L+3 (18.6%); H-1→L+2 (18.6%); H-2→L (18.4%); H-3→L+1 (18.4%); H-4→L+3 (6.9%); H-5→L (6.8%)
	7	3.92	316.30	0.0005	H→L+2 (18.6%); H-1→L+3 (18.6%); H-2→L+1 (18.4%); H-3→L (18.4%); H-4→L+2 (6.9%); H-5→L+1 (6.8%)
−0.01	4	3.34	371.53	0.0023	H→L+2 26.8%, H-1→L+3 26.3%, H-3→L 10.7%, H-2→L+1 10.2%, H→L 9.8%, H-1→L+1 6.0%
−0.015	3	2.98	416.19	0.0024	H→L 36.3%, H-1→L+1 21.2%, H→L+2 14.3%, H-1→L+3 9.7%
−0.02	3	2.67	466.09	0.0146	H-1→L+1 34.4%, H→L+2 31.7%, H-3→L 16.7%, H-2→L+3 9.9%
−0.025	3	2.1177	585.47	0.0047	H→L 69.2%, H-1→L+1 12.6%
−0.0275	3	1.9316	641.87	0.0075	H→L 75.7%, H-1→L+1 11.1%
−0.029	3	1.8238	679.81	0.0095	H→L 78.4%, H-1→L+1 10.7%

**Table 4:** Key electronic descriptors for selected excited states of B_6_C_6_N_6_ under various −F_y_ EEF at gas phase (HDI and EDI are dimensionless).

EEF	State	Sr (a.u.)	*D* (Å)	*t* (Å)	*H* (Å)	HDI	EDI	E_coul_ (eV)	Excitation Types
−0.01	S4	0.757	1.503	−0.893	3.490	7.43	4.99	4.425	LE
−0.015	S3	0.658	2.646	0.428	3.645	7.61	4.92	3.930	CT&LE
−0.02	S3	0.699	2.579	0.312	3.559	6.40	4.89	3.999	CT&LE
−0.025	S3	0.526	3.206	1.510	3.518	8.88	5.32	3.646	CT
−0.0275	S3	0.496	3.289	1.623	3.397	9.21	5.45	3.643	CT
−0.029	S3	0.485	3.324	1.659	3.349	9.31	5.48	3.637	CT

## Data Availability

The original contributions presented in this study are included in the article/Supplementary Material. Further inquiries can be directed to the corresponding author.

## Supplementary Materials

The following Supporting Information can be downloaded at https://www.mdpi.com/article/10.3390/molecules31203627/s1, Table S1: Structural parameters, polarizability, dipole moment and point group of B_6_C_6_N_6_ without EEF and of B_6_C_6_N_6_ under various EEFs; Figure S1: Optimized structures of B_6_C_6_N_6_ without EEF and of B_6_C_6_N_6_ under different EEFs; Figure S2. Changes in the longest axis length and dipole moment with the strength of EEF; Figure S3: LOL-π_in_ and LOL-π_out_ isosurfaces of B_6_C_6_N_6_ under different −F_y_ EEFs; Figure S4: Color-filled LOL maps at 1.2 Å above the B_6_C_6_N_6_ molecular plane under different −F_y_ EEFs; Figure S5: Color-filled map of electron localization function in the ring plane of B_6_C_6_N_6_ without EEF and of B_6_C_6_N_6_ under different −F_y_ EEFs; Figure S6: Electron density difference maps of B_6_C_6_N_6_ under different −F_y_ EEFs; Figure S7: Local integral curve of B_6_C_6_N_6_ under different −F_y_ EEFs; Table S2: Energies of HOMO, LUMO and HOMO-LUMO gap of B_6_C_6_N_6_ without EEF and under different −F_y_ EEFs; Figure S8: HOMO and LUMO isosurfaces of B_6_C_6_N_6_ without EEF and of B_6_C_6_N_6_ under different −F_y_ EEFs; Figure S9: Total density-of-states (TDOS) and partial density-of-states (PDOS) maps of B, C and N fragments of B_6_C_6_N_6_ without EEF and of B_6_C_6_N_6_ under different −F_y_ EEFs; Figure S10: UV-Vis spectra of B_6_C_6_N_6_ without EEF and of B_6_C_6_N_6_ under different −F_y_ EEFs; Figure S11: Charge transfer spectrum (CTS) of B_6_C_6_N_6_ without EEF and of B_6_C_6_N_6_ under different −F_y_ EEFs; Table S3: Excited energy, excited wavelength, oscillator strength (*f*) and main molecular orbital transitions of low-energy excited states of B_6_C_6_N_6_ without EEF and of B_6_C_6_N_6_ under various −F_y_ EEFs; Figure S12: Excited-state hole–electron analyses of B_6_C_6_N_6_ under various −F_y_ EEFs; Figure S13: Isosurface of orbital-weighted dual descriptor of B_6_C_6_N_6_ without EEF and of B_6_C_6_N_6_ under various −F_y_ EEFs; Supplementary Data S1: Optimized Cartesian coordinates.

## Abbreviations

The following abbreviations are used in this manuscript:

LMO	Localized molecular orbitals
ELF	Electron localization functional
LOL	Localized orbital locator
EDD	Electron density difference
CDC	Charge displacement curve
TDOS	Total density of states
PDOS	Partial density of states
CTS	Charge transfer spectra
OWDD	Orbital weighted dual descriptor
LEAE	Local electron attachment energy
