# Supplementary material for: Influence of External Electric Field on B6C6N6: Focusing on Structure, Electronic Structure, Spectra and Reactivity
Source: Molecules. 2026 Oct 12;31(20):3627. doi: 10.3390/molecules31203627 (PMC13707700; doi:10.3390/molecules31203627)
Supplement: Supplementary file 1 [file molecules-31-03627-s001.zip › Supplementary Data S1.pdf]

Supplementary Data S1

Optimized Cartesian coordinates of $B_6C_6N_6$ without EEF and of $B_6C_6N_6$ under various EEFs at the ωB97XD/def2-TZVP level. The values are given in Å.

EEF = 0 (charge = 0, spin multiplicity = 1)			
Atom	x	y	z
C	0.60640000	-3.81047000	0.00000000
C	-0.60640000	-3.81047000	0.00000000
C	-3.60316400	1.38007700	0.00000000
C	-2.99676400	2.43039300	0.00000000
C	3.60316400	1.38007700	0.00000000
C	2.99676400	2.43039300	0.00000000
B	1.81055900	3.30982500	0.00000000
B	-3.77167200	-0.08692300	0.00000000
B	1.96111300	-3.22290200	0.00000000
N	0.63772500	3.71824100	0.00000000
N	-3.53895400	-1.30683400	0.00000000
N	2.90122900	-2.41140700	0.00000000
N	-2.90122900	-2.41140700	0.00000000
N	3.53895400	-1.30683400	0.00000000
N	-0.63772500	3.71824100	0.00000000
B	-1.81055900	3.30982500	0.00000000
B	-1.96111300	-3.22290200	0.00000000
B	3.77167200	-0.08692300	0.00000000

EEF = 0.01 (charge = 0, spin multiplicity = 1)			
Atom	x	y	z
C	0.60582900	-3.98205500	0.00000000
C	-0.60582900	-3.98205500	0.00000000
C	-3.40184300	1.36792000	0.00000000
C	-2.90803700	2.47952100	0.00000000
C	3.40184300	1.36792000	0.00000000
C	2.90803700	2.47952100	0.00000000
B	1.75862600	3.39464000	0.00000000
B	-3.59373200	-0.10122900	0.00000000
B	1.93493600	-3.34619900	0.00000000
N	0.64796200	3.97074600	0.00000000
N	-3.32437800	-1.30792200	0.00000000
N	2.83105200	-2.47542400	0.00000000
N	-2.83105200	-2.47542400	0.00000000
N	3.32437800	-1.30792200	0.00000000
N	-0.64796200	3.97074600	0.00000000
B	-1.75862600	3.39464000	0.00000000
B	-1.93493600	-3.34619900	0.00000000

B	3.59373200	-0.10122900	0.00000000

EEF = 0.015 (charge = 0, spin multiplicity = 1)			
Atom	x	y	z
C	0.60592800	-4.13318200	0.00000000
C	-0.60592800	-4.13318200	0.00000000
C	-3.19962000	1.35599400	0.00000000
C	-2.78592700	2.50425500	0.00000000
C	3.19962000	1.35599400	0.00000000
C	2.78592700	2.50425500	0.00000000
B	1.69578800	3.48042600	0.00000000
B	-3.39505800	-0.11157600	0.00000000
B	1.90847100	-3.44899900	0.00000000
N	0.65888800	4.20008600	0.00000000
N	-3.15249000	-1.32458100	0.00000000
N	2.75533300	-2.52242300	0.00000000
N	-2.75533300	-2.52242300	0.00000000
N	3.15249000	-1.32458100	0.00000000
N	-0.65888800	4.20008600	0.00000000
B	-1.69578800	3.48042600	0.00000000
B	-1.90847100	-3.44899900	0.00000000
B	3.39505800	-0.11157600	0.00000000

EEF = 0.02 (charge = 0, spin multiplicity = 1)			
Atom	x	y	z
C	0.60651600	-4.35271200	0.00000000
C	-0.60651600	-4.35271200	0.00000000
C	-2.85690700	1.33723100	0.00000000
C	-2.53350600	2.52044800	0.00000000
C	2.85690700	1.33723100	0.00000000
C	2.53350600	2.52044800	0.00000000
B	1.57603400	3.61481400	0.00000000
B	-3.05133500	-0.12608300	0.00000000
B	1.86102600	-3.58952700	0.00000000
N	0.68061500	4.53328700	0.00000000
N	-2.88628900	-1.35512000	0.00000000
N	2.62377500	-2.58233800	0.00000000
N	-2.62377500	-2.58233800	0.00000000
N	2.88628900	-1.35512000	0.00000000
N	-0.68061500	4.53328700	0.00000000
B	-1.57603400	3.61481400	0.00000000
B	-1.86102600	-3.58952700	0.00000000
B	3.05133500	-0.12608300	0.00000000

EEF = 0.025 (charge = 0, spin multiplicity = 1)

Atom	x	y	z
C	0.60791300	-4.59663500	0.00000000
C	-0.60791300	-4.59663500	0.00000000
C	-2.40431900	1.31635000	0.00000000
C	-2.15648000	2.52623600	0.00000000
C	2.40431900	1.31635000	0.00000000
C	2.15648000	2.52623600	0.00000000
B	1.41502300	3.76409600	0.00000000
B	-2.59324200	-0.14012700	0.00000000
B	1.78738300	-3.72870800	0.00000000
N	0.71334800	4.86992400	0.00000000
N	-2.53830800	-1.38537900	0.00000000
N	2.42595000	-2.62575800	0.00000000
N	-2.42595000	-2.62575800	0.00000000
N	2.53830800	-1.38537900	0.00000000
N	-0.71334800	4.86992400	0.00000000
B	-1.41502300	3.76409600	0.00000000
B	-1.78738300	-3.72870800	0.00000000
B	2.59324200	-0.14012700	0.00000000

EEF = 0.0275 (charge = 0, spin multiplicity = 1)			
Atom	x	y	z
C	0.60909100	-4.68618200	0.00005900
C	-0.60872400	-4.68606400	0.00005700
C	-2.23391800	1.31212800	-0.00006100
C	-2.01655600	2.53272600	-0.00001800
C	2.23357200	1.31156100	-0.00006500
C	2.01568000	2.53206600	-0.00002400
B	1.35899600	3.81228200	0.00002000
B	-2.40961700	-0.14220200	-0.00005500
B	1.75274000	-3.77475200	0.00003100
N	0.72526600	4.97071400	0.00006800
N	-2.39208000	-1.39284400	-0.00004000
N	2.33010600	-2.63196700	-0.00000200
N	-2.32929700	-2.63150500	-0.00000800
N	2.39315400	-1.39332000	-0.00003500
N	-0.72587000	4.97121700	0.00007400
B	-1.36043500	3.81323000	0.00002700
B	-1.75221600	-3.77442300	0.00002600
B	2.41010700	-0.14266700	-0.00005500

EEF = 0.029 (charge = 0, spin multiplicity = 1)			
Atom	x	y	z
C	0.60960800	-4.73311400	0.00000000
C	-0.60960800	-4.73311400	0.00000000

C	-2.14959400	1.31099500	0.00000000
C	-1.94984300	2.53761700	0.00000000
C	2.14959400	1.31099500	0.00000000
C	1.94984300	2.53761700	0.00000000
B	1.33240200	3.83515100	0.00000000
B	-2.31443800	-0.14260100	0.00000000
B	1.73183100	-3.79710300	0.00000000
N	0.73215300	5.01788400	0.00000000
N	-2.31467400	-1.39615900	0.00000000
N	2.27236000	-2.63267000	0.00000000
N	-2.27236000	-2.63267000	0.00000000
N	2.31467400	-1.39615900	0.00000000
N	-0.73215300	5.01788400	0.00000000
B	-1.33240200	3.83515100	0.00000000
B	-1.73183100	-3.79710300	0.00000000
B	2.31443800	-0.14260100	0.00000000

EEF = −0.01 (charge = 0, spin multiplicity = 1)			
Atom	x	y	z
C	0.60770300	-3.74387000	0.00000000
C	-0.60770300	-3.74387000	0.00000000
C	-3.65010500	1.37281400	0.00000000
C	-2.99741500	2.39638900	0.00000000
C	3.65010500	1.37281400	0.00000000
C	2.99741500	2.39638900	0.00000000
B	1.82762900	3.30597500	0.00000000
B	-3.79585000	-0.08396000	0.00000000
B	1.97260900	-3.17183000	0.00000000
N	0.63209200	3.63139300	0.00000000
N	-3.67726800	-1.33764700	0.00000000
N	2.91534900	-2.36926400	0.00000000
N	-2.91534900	-2.36926400	0.00000000
N	3.67726800	-1.33764700	0.00000000
N	-0.63209200	3.63139300	0.00000000
B	-1.82762900	3.30597500	0.00000000
B	-1.97260900	-3.17183000	0.00000000
B	3.79585000	-0.08396000	0.00000000

EEF = −0.015 (charge = 0, spin multiplicity = 1)			
Atom	x	y	z
C	0.60870600	-3.76681400	0.00000000
C	-0.60870600	-3.76681400	0.00000000
C	-3.60714400	1.36188200	0.00000000
C	-2.96673900	2.39547900	0.00000000

C	3.60714400	1.36188200	0.00000000
C	2.96673900	2.39547900	0.00000000
B	1.82945600	3.34845200	0.00000000
B	-3.74774000	-0.08730400	0.00000000
B	1.97228500	-3.18729500	0.00000000
N	0.62971600	3.65801100	0.00000000
N	-3.69580000	-1.35784800	0.00000000
N	2.89736200	-2.36456400	0.00000000
N	-2.89736200	-2.36456400	0.00000000
N	3.69580000	-1.35784800	0.00000000
N	-0.62971600	3.65801100	0.00000000
B	-1.82945600	3.34845200	0.00000000
B	-1.97228500	-3.18729500	0.00000000
B	3.74774000	-0.08730400	0.00000000

EEF = −0.02 (charge = 0, spin multiplicity = 1)			
Atom	x	y	z
C	0.61016000	-3.86034300	0.00000000
C	-0.61016000	-3.86034300	0.00000000
C	-3.49878900	1.35234800	0.00000000
C	-2.90546800	2.41704500	0.00000000
C	3.49878900	1.35234800	0.00000000
C	2.90546800	2.41704500	0.00000000
B	1.82734400	3.43820200	0.00000000
B	-3.63180500	-0.08913300	0.00000000
B	1.96433900	-3.25508200	0.00000000
N	0.62717000	3.75067900	0.00000000
N	-3.63819200	-1.37296200	0.00000000
N	2.84329700	-2.38075500	0.00000000
N	-2.84329700	-2.38075500	0.00000000
N	3.63819200	-1.37296200	0.00000000
N	-0.62717000	3.75067900	0.00000000
B	-1.82734400	3.43820200	0.00000000
B	-1.96433900	-3.25508200	0.00000000
B	3.63180500	-0.08913300	0.00000000

EEF = −0.025 (charge = 0, spin multiplicity = 1)			
Atom	x	y	z
C	0.61235600	-4.03269900	0.00000000
C	-0.61235600	-4.03269900	0.00000000
C	-3.30878900	1.34927800	0.00000000
C	-2.80306400	2.46299500	0.00000000
C	3.30878900	1.34927800	0.00000000
C	2.80306400	2.46299500	0.00000000
B	1.82109300	3.57651000	0.00000000

B	-3.42526900	-0.08540500	0.00000000
B	1.94811700	-3.38366400	0.00000000
N	0.62392200	3.91197000	0.00000000
N	-3.47304600	-1.37864800	0.00000000
N	2.73605300	-2.42033800	0.00000000
N	-2.73605300	-2.42033800	0.00000000
N	3.47304600	-1.37864800	0.00000000
N	-0.62392200	3.91197000	0.00000000
B	-1.82109300	3.57651000	0.00000000
B	-1.94811700	-3.38366400	0.00000000
B	3.42526900	-0.08540500	0.00000000

EEF = −0.0275 (charge = 0, spin multiplicity = 1)			
Atom	x	y	z
C	0.61393900	-4.14916100	0.00000000
C	-0.61393900	-4.14916000	0.00000000
C	-3.17482900	1.35246400	0.00000000
C	-2.73088200	2.49538600	0.00000000
C	3.17482900	1.35246400	0.00000000
C	2.73088200	2.49538600	0.00000000
B	1.81567400	3.66295700	0.00000000
B	-3.27428100	-0.07962000	0.00000000
B	1.93856200	-3.47532500	0.00000000
N	0.62161000	4.01967700	0.00000000
N	-3.32790100	-1.37646400	0.00000000
N	2.65209300	-2.44991500	0.00000000
N	-2.65209300	-2.44991500	0.00000000
N	3.32790100	-1.37646400	0.00000000
N	-0.62161000	4.01967700	0.00000000
B	-1.81567400	3.66295700	0.00000000
B	-1.93856200	-3.47532500	0.00000000
B	3.27428100	-0.07962000	0.00000000

EEF = −0.029 (charge = 0, spin multiplicity = 1)			
Atom	x	y	z
C	-0.60918500	4.73251100	0.00072900
C	0.61007700	4.73205700	-0.00085200
C	2.14965300	-1.31519600	0.00032700
C	1.95202000	-2.54256100	0.00056500
C	-2.15329400	-1.30702700	0.00072100
C	-1.95079700	-2.53306300	-0.00062900
B	-1.32272400	-3.82587300	-0.00920300
B	2.31150500	0.13824900	-0.00160500
B	-1.73529800	3.80059500	0.00148300
N	-0.72548100	-5.00965800	0.00103300

N	2.31066900	1.39214500	-0.00021100
N	-2.27646200	2.63674000	0.00197200
N	2.26913000	2.62820000	-0.00270100
N	-2.31918600	1.40012100	0.00306400
N	0.73831700	-5.02524900	0.00113200
B	1.34062600	-3.84248900	0.00331900
B	1.72984100	3.79380100	-0.00199500
B	-2.31941000	0.14669500	0.00285000
