# Supplementary material for: Influence of External Electric Field on B6C6N6: Focusing on Structure, Electronic Structure, Spectra and Reactivity
Source: Molecules. 2026 Oct 12;31(20):3627. doi: 10.3390/molecules31203627 (PMC13707700; doi:10.3390/molecules31203627)
Supplement: Supplementary file 1 [file molecules-31-03627-s001.zip › supporting information.v7.pdf]

Supporting information

Table S1(a). Structural parameters, polarizability, dipole moment and point group of $B_6C_6N_6$ without EEF and of $B_6C_6N_6$ under various $-F_y$ EEFs

EEF	Length of the three sides (Å)			D (Å)	cav (Å)	MPP (Å)	Polarizability (α)	Dipole Moment	Point group
0	11.023	10.779	3.480	7.631	4.065	0	241.518	0	D_{3h}
-0.01	10.667	11.203	3.480	8.051	3.710	0	246.937	10.569	C_{2v}
-0.015	10.270	11.583	3.480	8.429	3.314	0	254.597	17.137	C_{2v}
-0.02	9.583	12.136	3.480	8.979	2.629	0	267.232	25.706	C_{2v}
-0.025	8.666	12.717	3.480	9.558	1.728	0	286.245	36.443	C_{2v}
-0.0275	8.300	12.908	3.480	9.749	1.376	0.000046	297.075	41.821	C_{2v}
-0.029	8.109	13.001	3.480	9.843	1.201	0	304.005	45.068	C_{2v}

Note: Length of the three sides, *D*, cav, MPP are the length, width and height of $B_6C_6N_6$ under various EEF strengths, the length of the longer axis, cavity diameter, molecular planarity parameter, respectively. Dipole moments are in Debye.

Table S1(b). Structural parameters, polarizability, dipole moment and point group of $B_6C_6N_6$ under various $+F_y$ EEF strengths

EEF	Length of the three sides (Å)			*D* (Å)	cav (Å)	MPP (Å)	Polarizability (α)	Dipole Moment	Point group
0	11.023	10.779	3.480	7.631	4.065	0	241.518	0	D_{3h}
0.01	11.072	10.625	3.480	7.813	4.016	0	246.404	9.744	C_{2v}
0.015	10.975	10.675	3.480	7.793	4.019	0	253.194	14.727	C_{2v}
0.02	10.744	10.861	3.480	7.711	3.788	0	265.496	20.154	C_{2v}
0.025	10.331	11.195	3.480	8.040	3.373	0	290.352	26.809	C_{2v}
0.0275	10.029	11.419	3.480	8.262	3.070	0	316.394	31.180	C_{2v}
0.029	8.111	13.011	3.493	9.850	1.201	0.0027	304.069	45.086	C1

Note: Length of the three sides, *D*, cav, MPP are the length, width and height of $B_6C_6N_6$ under various EEF strengths, the length of the longer axis, cavity diameter, molecular planarity parameter, respectively. Dipole moments are in Debye.

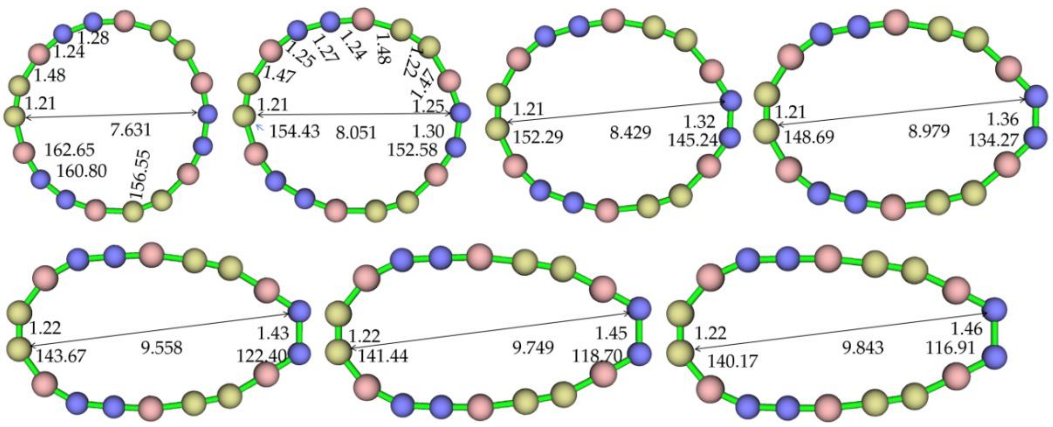

Figure S1(a). Optimized structures of $B_6C_6N_6$ without EEF and of $B_6C_6N_6$ under different $-F_y$ EEFs (a) 0, (b) −0.01, (c) −0.015, (d) −0.02, (e) −0.025, (f) −0.0275, and (g) −0.029 a.u. The arrow indicates the molecular size *D* (the length of the longest axis, in Å). Other values are bond lengths (in Å) and bond angles (in °).

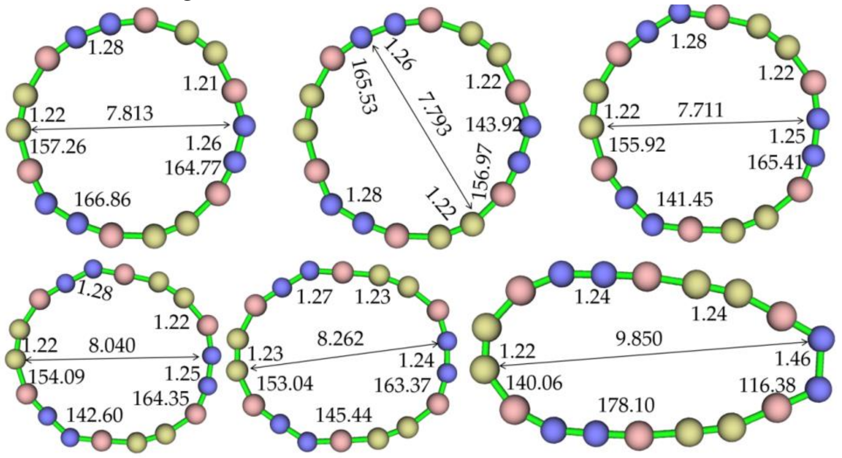

Figure S1(b). Optimized structures of $B_6C_6N_6$ under different $+F_y$ EEFs (a) 0.01, (b) 0.015, (c) 0.02, (d) 0.025, (e) 0.0275, and (f) 0.029 a.u. The arrow indicates the molecular size D (in Å).

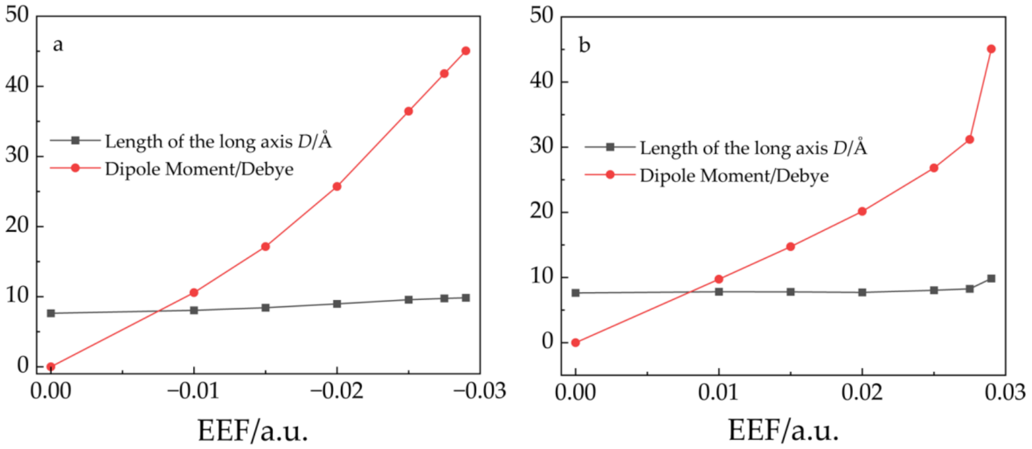

Figure S2. Changes in the length of the long axis length (*D*, in Å) and dipole moment (in Debye) with the strength of $-F_y$ EEF (a) and $+F_y$ EEF (b)

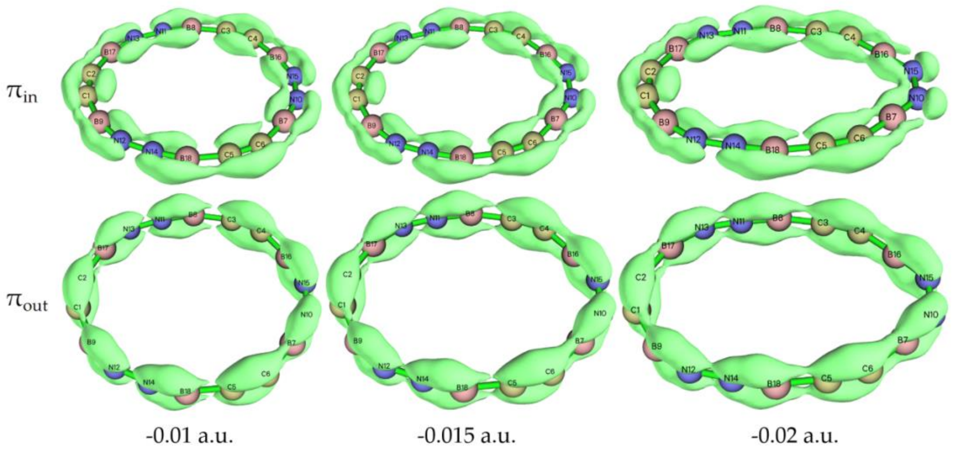

Figure S3(a). LOL-π_{in} and LOL-π_{out} isosurfaces of $B_6C_6N_6$ under different $-F_y$ EEFs (−0.01 a.u.,

−0.015 a.u. and −0.02 a.u.). Isovalue = 0.4 a.u.

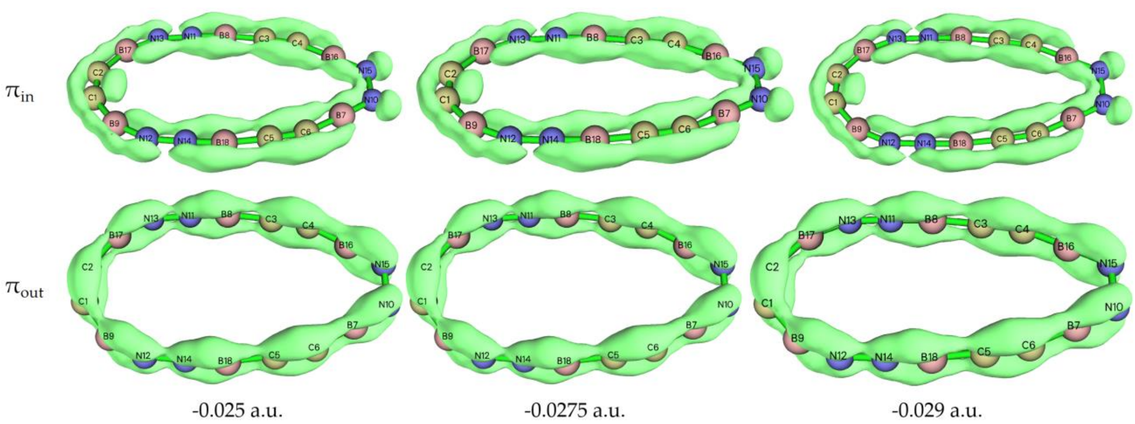

Figure S3(b). LOL-π_{in} and LOL-π_{out} isosurfaces of $B_6C_6N_6$ under different $-F_y$ EEFs (−0.025 a.u., −0.0275 a.u. and −0.029 a.u.). Isovalue = 0.4 a.u.

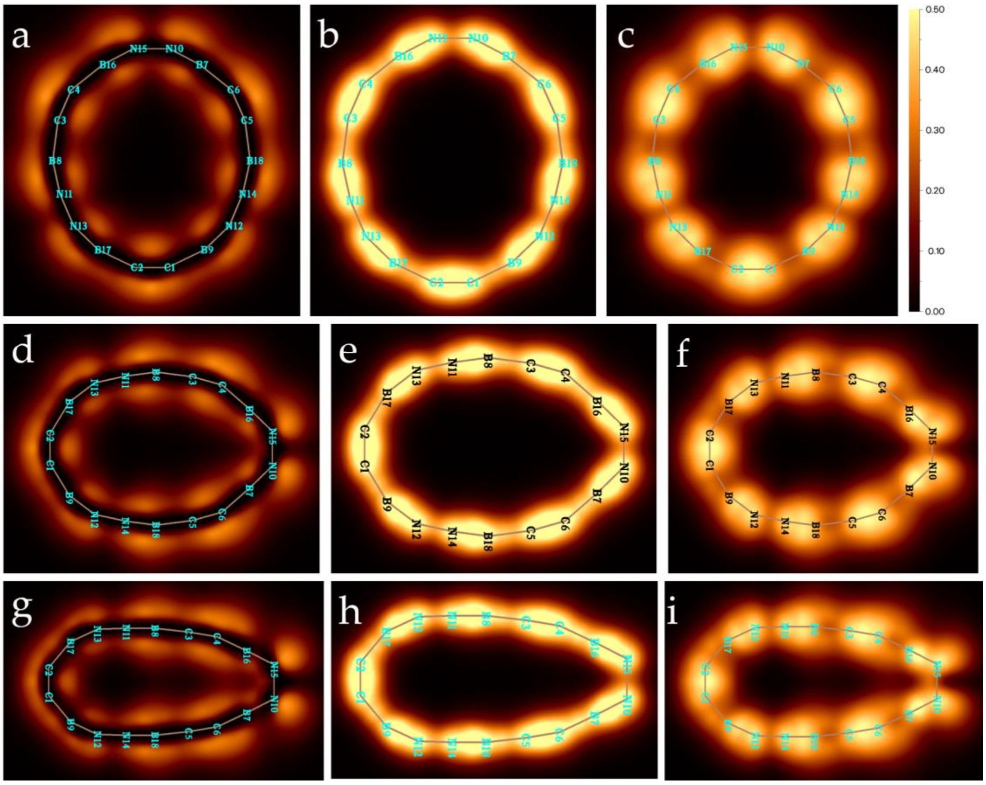

Figure S4. Color-filled LOL maps at 1.2 Å above the $B_6C_6N_6$ molecular plane under different $-F_y$ EEFs (a–c: −0.01 a.u., d–f: −0.025 a.u., g–i: −0.029 a.u.). The first column corresponds to LOL-π_{in}; the second column to LOL-π_{out}; the third column to total LOL-π.

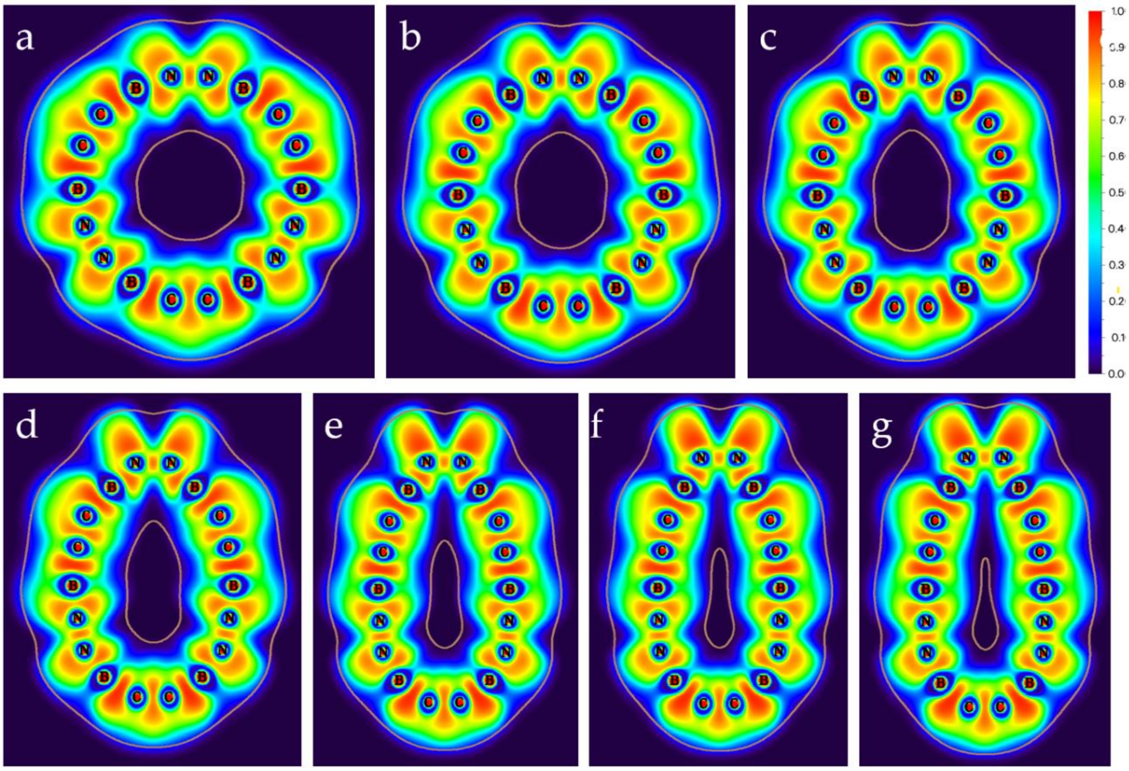

Figure S5(a). Color-filled map of electron localization function in the ring plane of $B_6C_6N_6$ without EEF and of $B_6C_6N_6$ under different $-F_y$ EEFs (a) 0, (b) −0.01, (c) −0.015, (d) −0.02, (e) −0.025, (f) −0.0275, and (g) −0.029 a.u.. The brown contour line portrays electron density of 0.001 a.u.

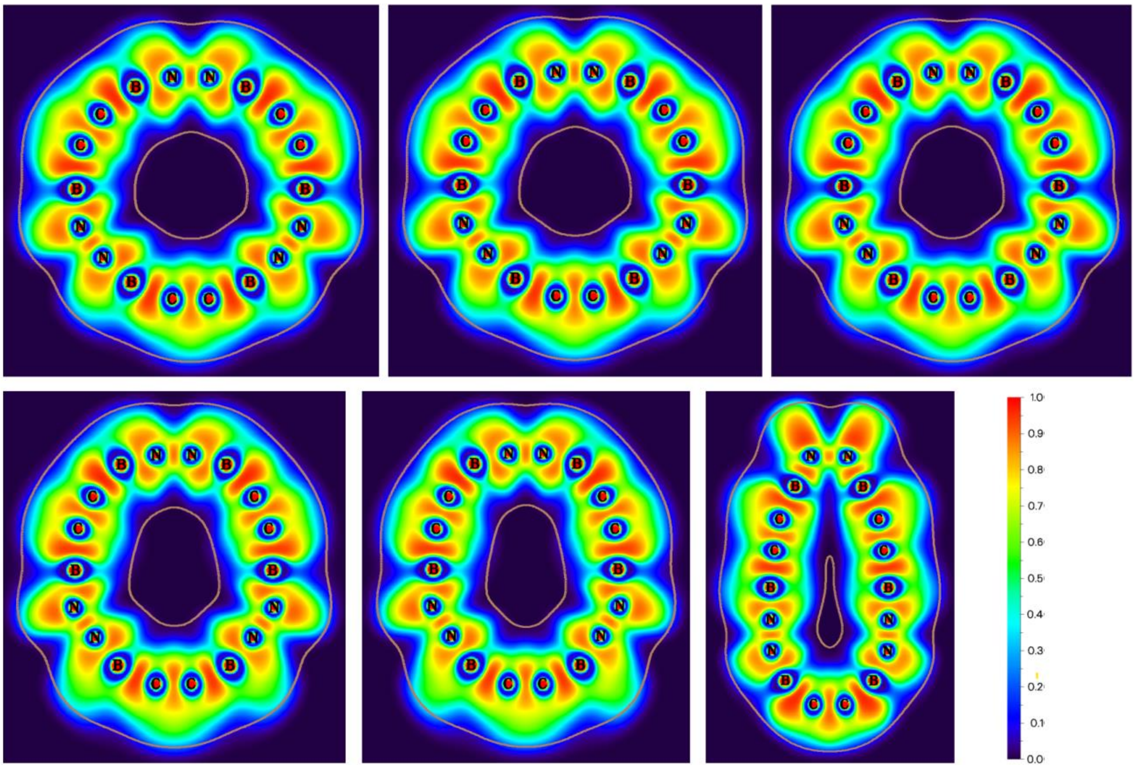

Figure S5(b). Color-filled map of electron localization function in the ring plane of $B_6C_6N_6$ under different $+F_y$ EEFs (a) 0.01, (b) 0.015, (c) 0.02, (d) 0.025, (e) 0.0275, and (f) 0.029 a.u. The

brown contour line portrays electron density of 0.001 a.u.

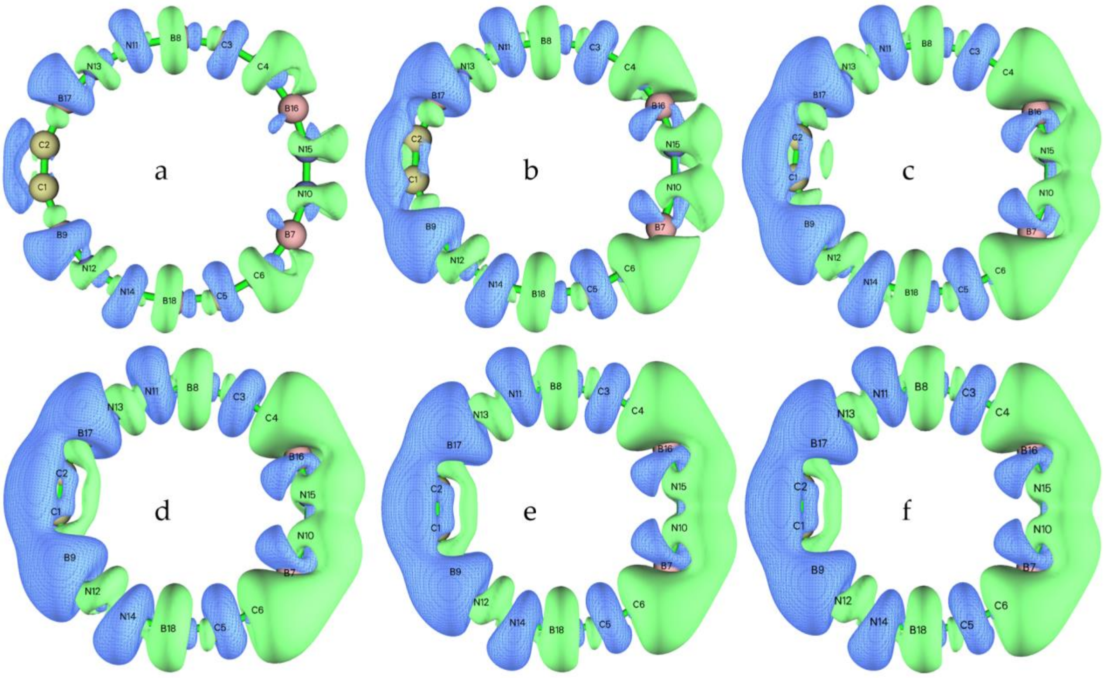

Figure S6. Electron density difference maps of $B_6C_6N_6$ under different $-F_y$ EEFs (a: −0.01 a.u.; b: −0.015 a.u.; c: −0.02 a.u.; d: −0.025 a.u.; e: −0.0275 a.u.; f: −0.029 a.u.). Isovalue = 0.001 a.u. The green isosurface represents regions of increased electron density upon applying the electric field, while the blue isosurface corresponds to regions with decreased electron density.

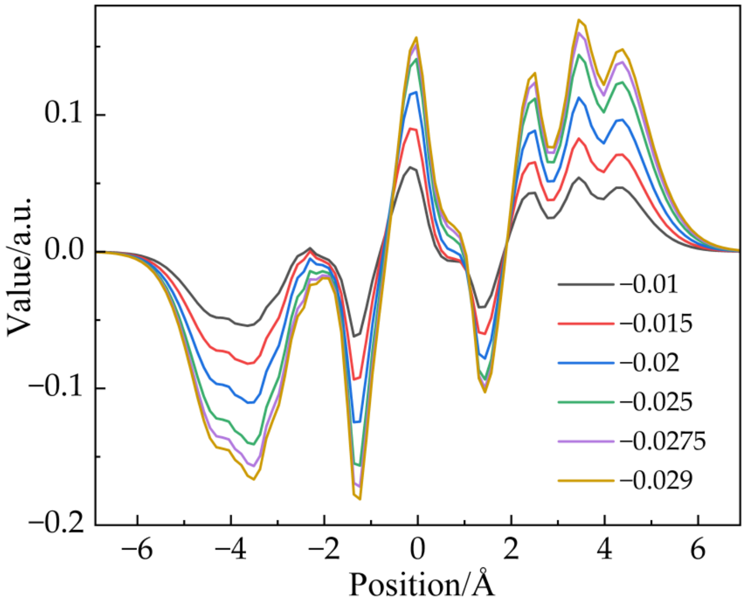

Figure S7. Local integral curve of $B_6C_6N_6$ under different $-F_y$ EEFs (The values in the figure denote the EEF strength in a.u.).

Table S2. Energies of HOMO, LUMO and HOMO-LUMO gap of $B_6C_6N_6$ without EEF and of $B_6C_6N_6$ under different $-F_y$ EEFs

EEF (a.u.)	E_{HOMO} (eV)	E_{LUMO} (eV)	E_{gap} (eV)
0	−8.63	−0.82	7.81
-0.01	−8.31	−1.26	7.05
-0.015	−8.17	−1.56	6.61
-0.02	−8.07	−1.97	6.10
-0.025	−8.05	−2.69	5.36
-0.0275	−8.05	−2.91	5.14
-0.029	−8.06	−3.02	5.03

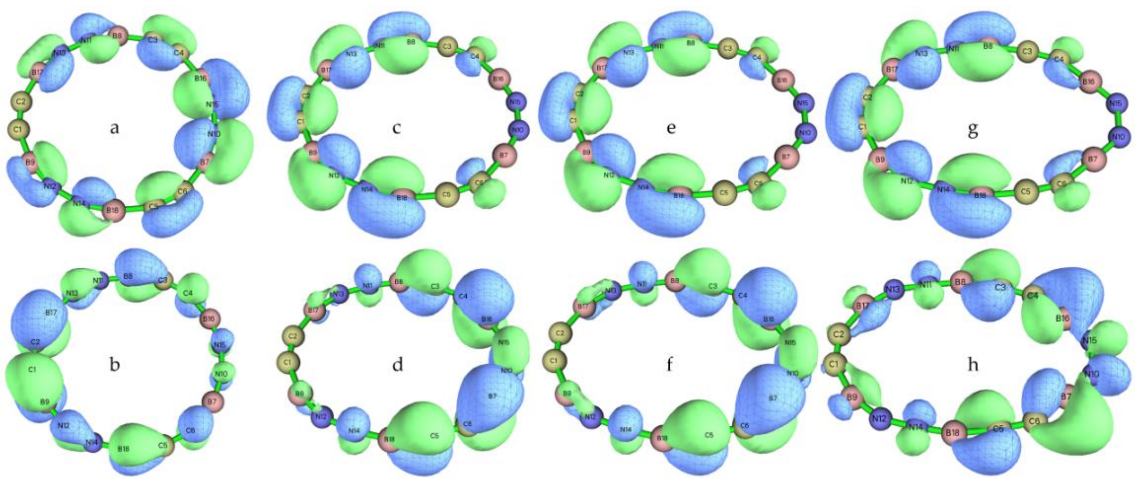

Figure S8(a). HOMO and LUMO isosurfaces of $B_6C_6N_6$ without EEF and of $B_6C_6N_6$ under different $-F_y$ EEFs: 0 (a and b), −0.01 (c and d), −0.015 (e and f), −0.02 a.u. (g and h). The first row are HOMO isosurfaces, and the second row are LUMO isosurfaces. Isovalue = 0.03 a.u. The positive and negative orbital phases are distinguished by the two colors.

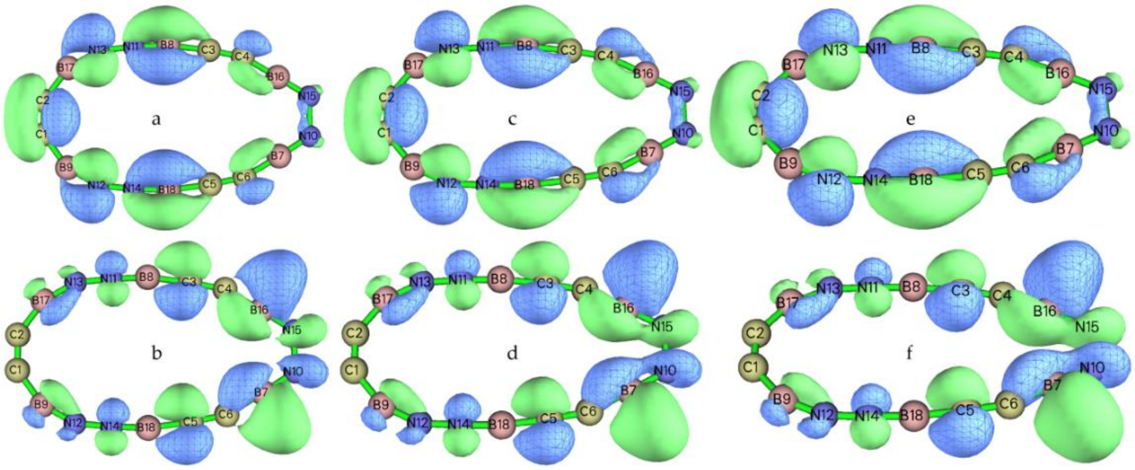

Figure S8(b). HOMO and LUMO isosurfaces of of $B_6C_6N_6$ under different $-F_y$ EEFs: −0.025 (a and b), −0.0275 (c and d), −0.029 a.u. (e and f). The first row are HOMO isosurfaces, and the second row are LUMO isosurfaces. Isovalue = 0.03 a.u. The positive and negative orbital phases are distinguished by the two colors.

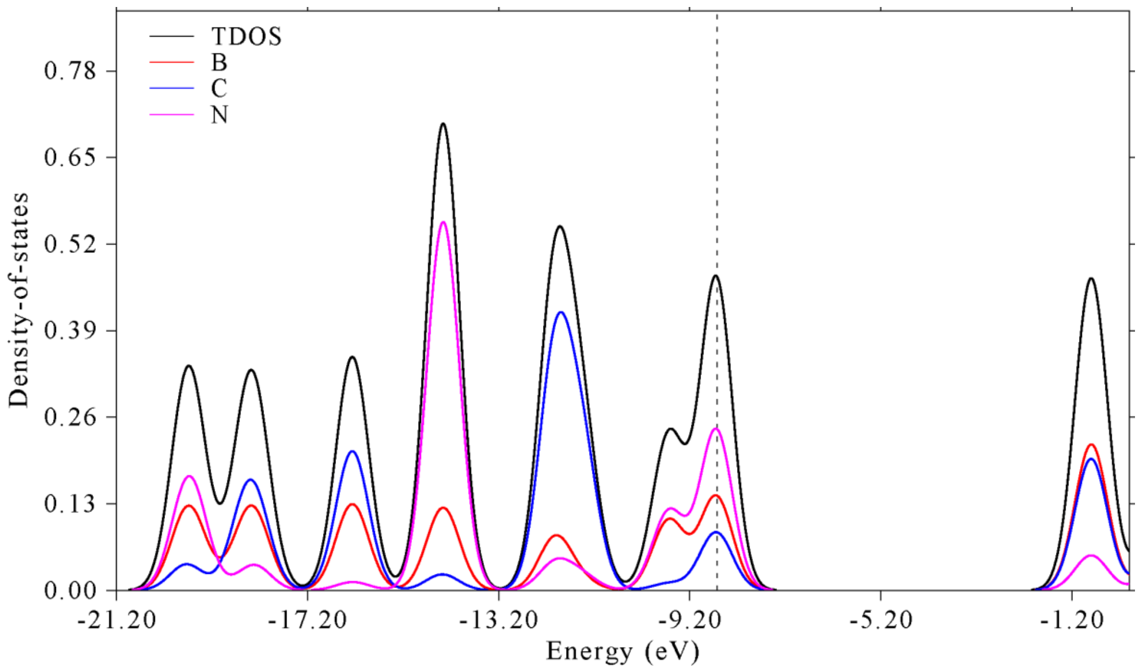

Figure S9(a). Total density-of-states (TDOS) and partial density-of-states (PDOS) maps of B, C and N fragments of $B_6C_6N_6$ without EEF, with full width at half maximum (FWHM) 0.8 eV.

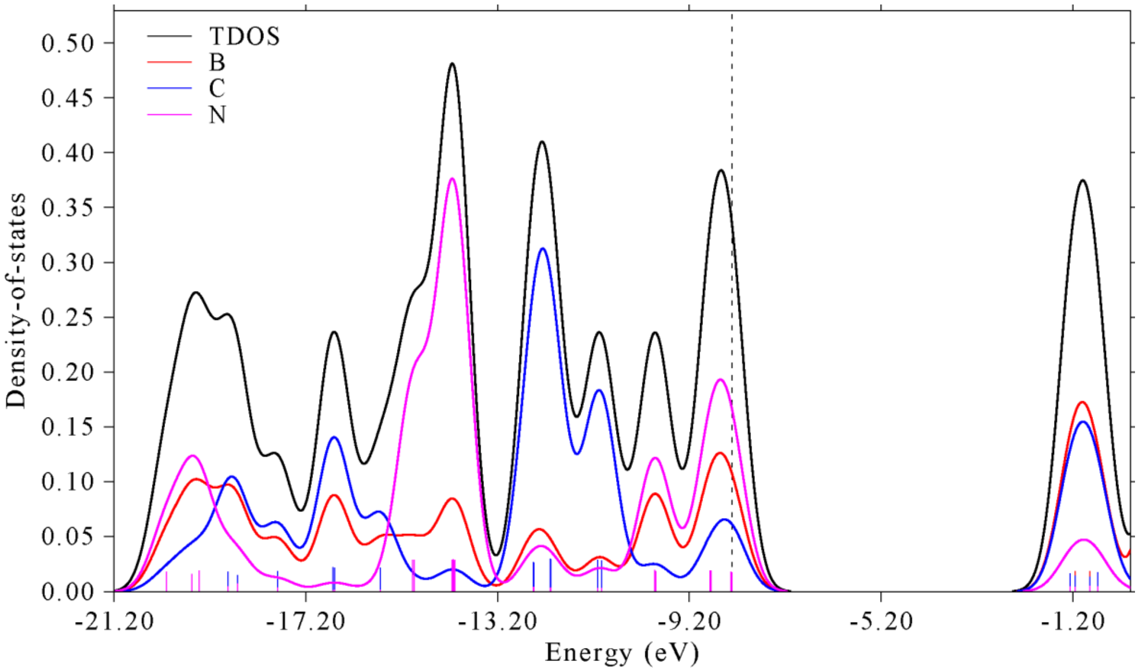

Figure S9(b). Total density-of-states (TDOS) and partial density-of-states (PDOS) maps of B, C and N fragments of $B_6C_6N_6$ under $-F_y$ EEF of −0.01 a.u., with full width at half maximum (FWHM) 0.8 eV.

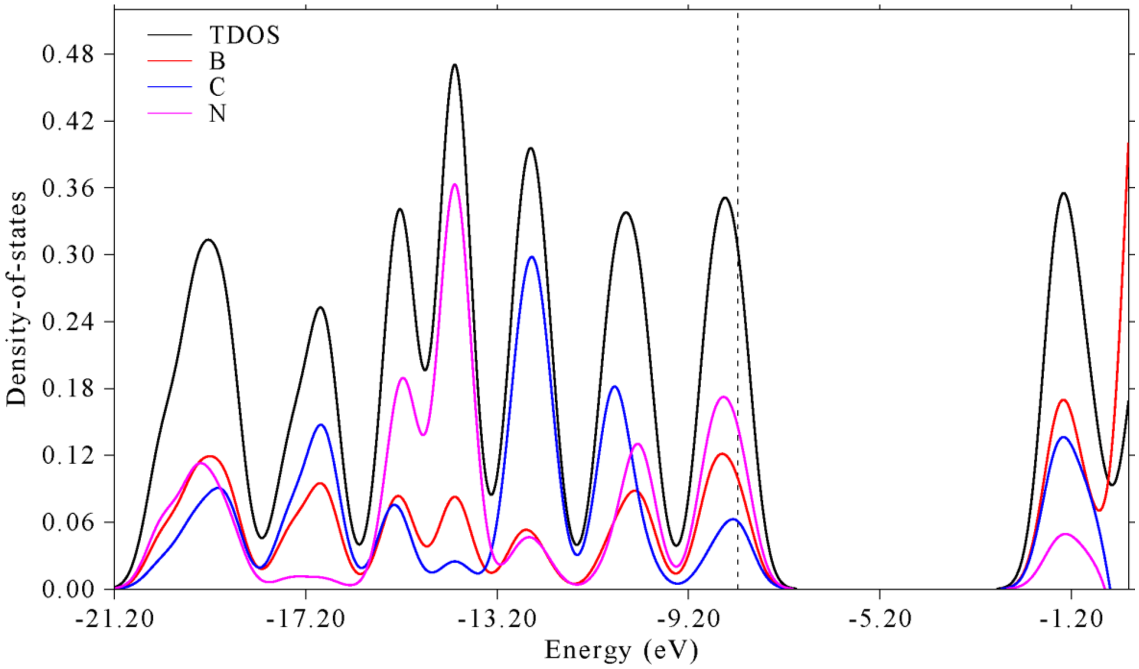

Figure S9(c). Total density-of-states (TDOS) and partial density-of-states (PDOS) maps of B, C and N fragments of $B_6C_6N_6$ under $-F_y$ EEF of −0.015 a.u., with full width at half maximum (FWHM) 0.8 eV.

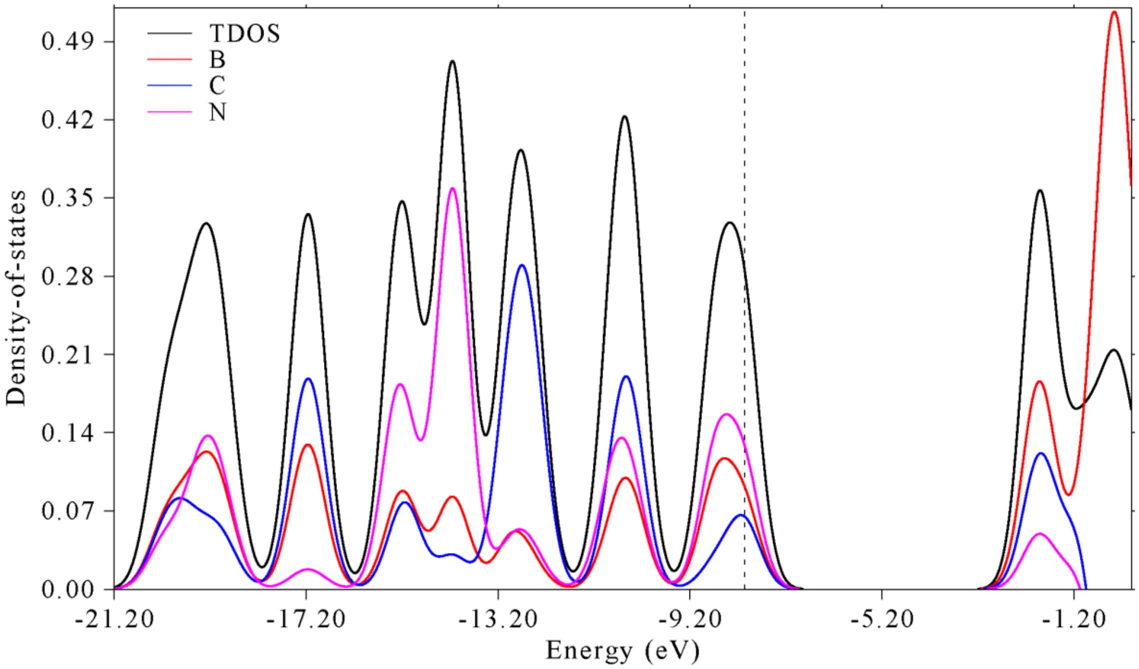

Figure S9(d). Total density-of-states (TDOS) and partial density-of-states (PDOS) maps of B, C and N fragments of $B_6C_6N_6$ under $-F_y$ EEF of −0.02 a.u., with full width at half maximum (FWHM) 0.8 eV.

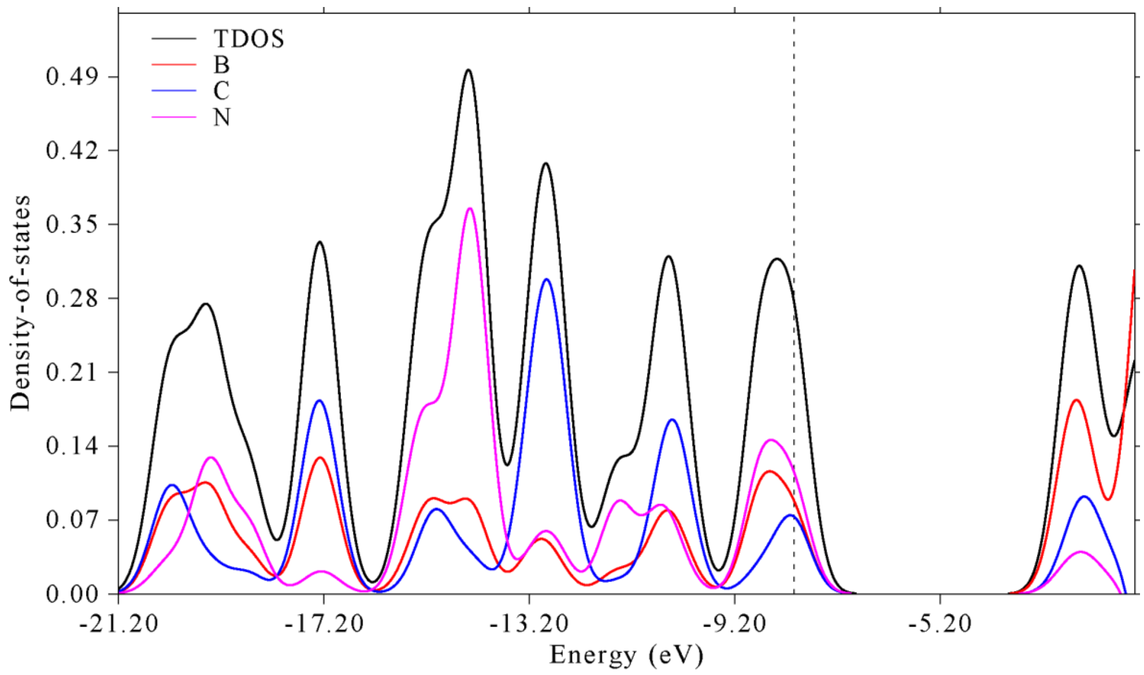

Figure S9(e). Total density-of-states (TDOS) and partial density-of-states (PDOS) maps of B, C and N fragments of $B_6C_6N_6$ under $-F_y$ EEF of −0.025 a.u., with full width at half maximum (FWHM) 0.8 eV.

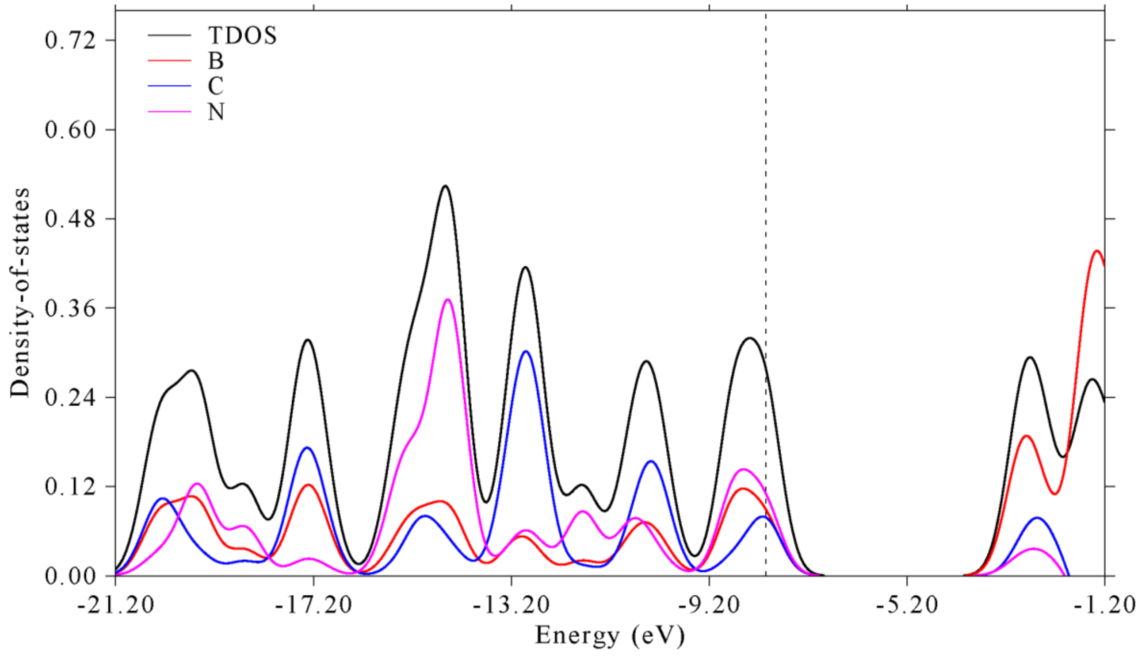

Figure S9(f). Total density-of-states (TDOS) and partial density-of-states (PDOS) maps of B, C and N fragments of $B_6C_6N_6$ under $-F_y$ EEF of −0.0275 a.u., with full width at half maximum (FWHM) 0.8 eV.

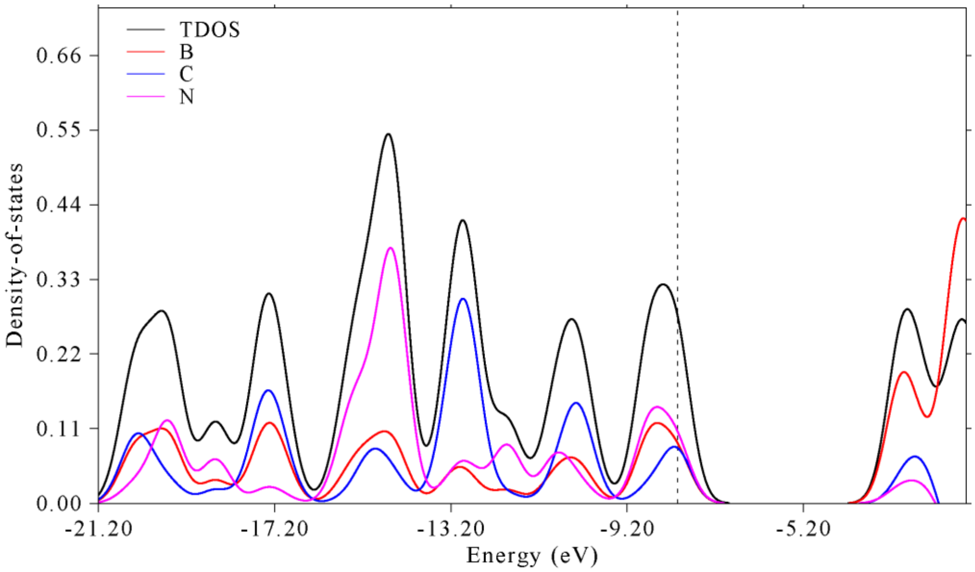

Figure S9(g). Total density-of-states (TDOS) and partial density-of-states (PDOS) maps of B, C and N fragments of $B_6C_6N_6$ under −F_y EEF of −0.029 a.u., with full width at half maximum (FWHM) 0.8 eV.

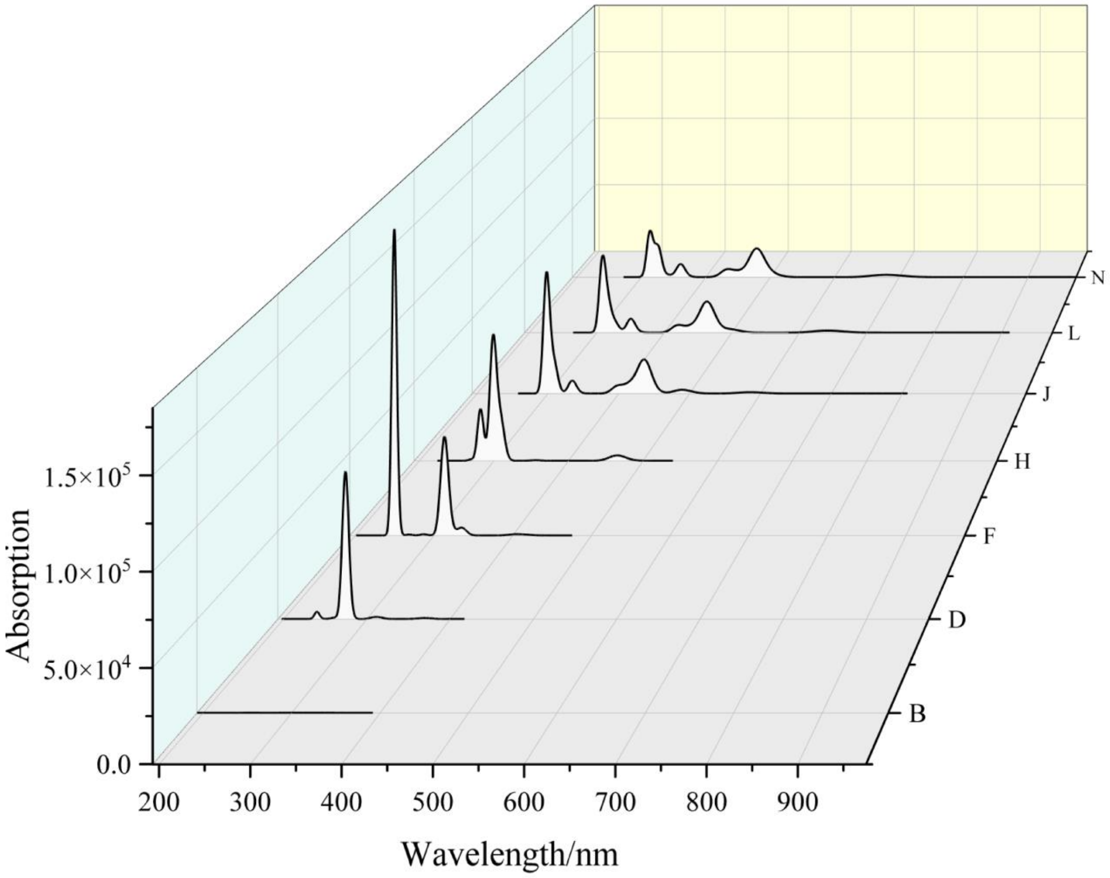

Figure S10(a). UV-Vis spectra of $B_6C_6N_6$ without EEF and of $B_6C_6N_6$ under different −F_y EEFs (B) 0, (D) −0.01, (F) −0.015, (H) −0.02, (J) −0.025, (L) −0.0275, and (N) −0.029 a.u. The full width at half maximum (FWHM) is 0.3 eV.

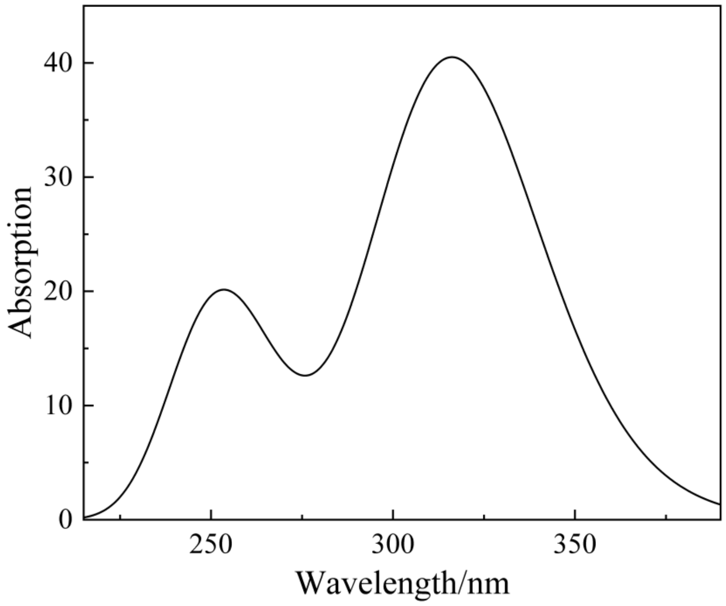

Figure S10(b). UV-Vis spectra of $B_6C_6N_6$ without EEF. The full width at half maximum (FWHM) is 0.3 eV.

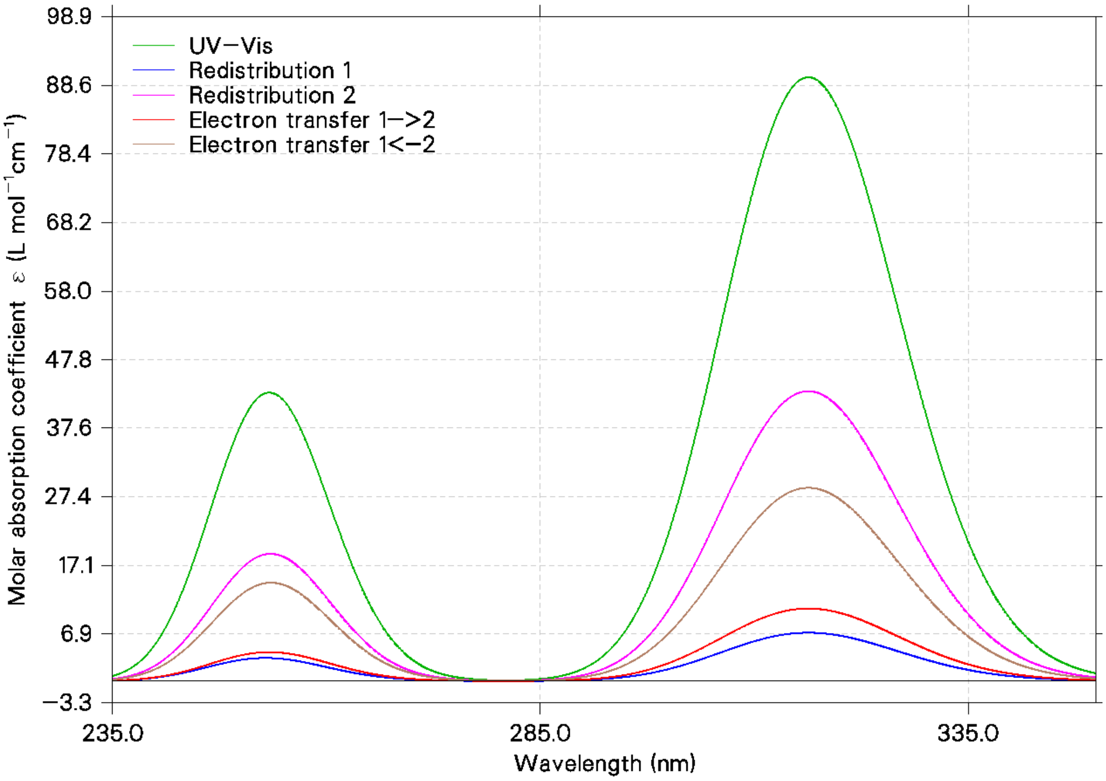

Figure S11(a). Charge transfer spectrum (CTS) of $B_6C_6N_6$ without EEF based on Hirshfeld partition (Fragments 1 and 2 are C and B&N, respectively. The full width at half maximum (FWHM) is 0.3 eV. The absorption band range is at 235–350 nm.)

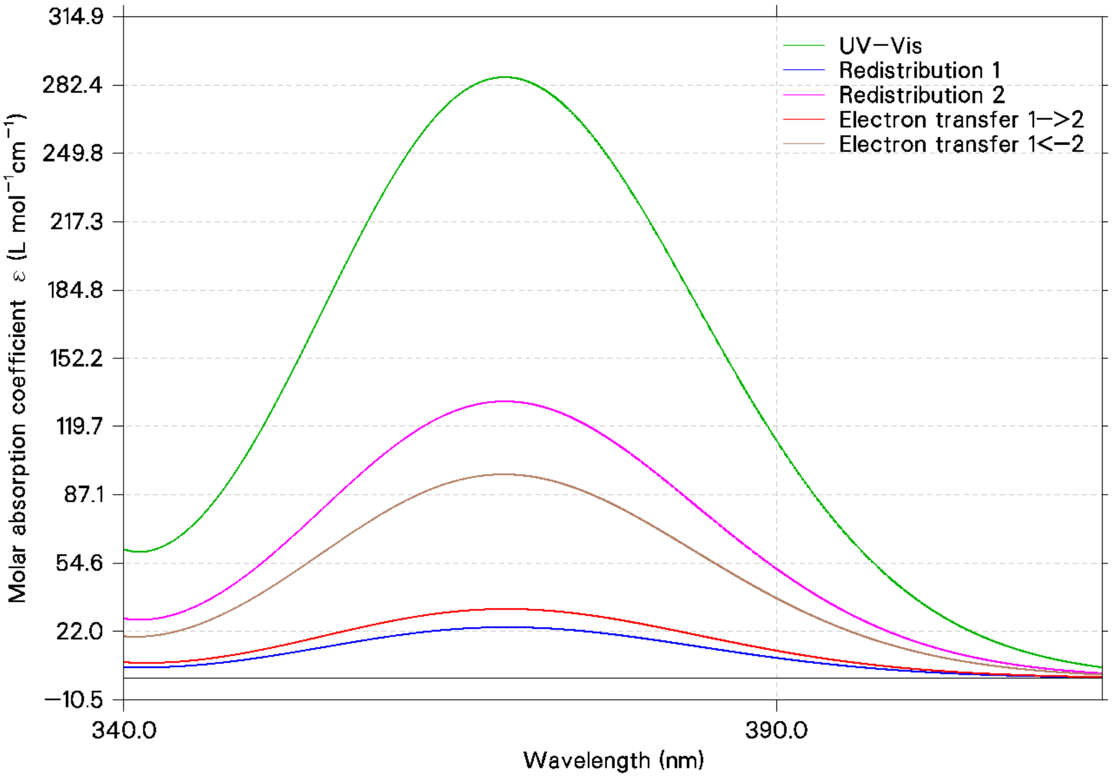

Figure S11(b). Charge transfer spectrum (CTS) of $B_6C_6N_6$ under $-F_y$ EEF of −0.01 a.u. based on Hirshfeld partition (Fragments 1 and 2 are C and B&N, respectively. The full width at half maximum (FWHM) is 0.3 eV. The absorption band range is at 340–415 nm.)

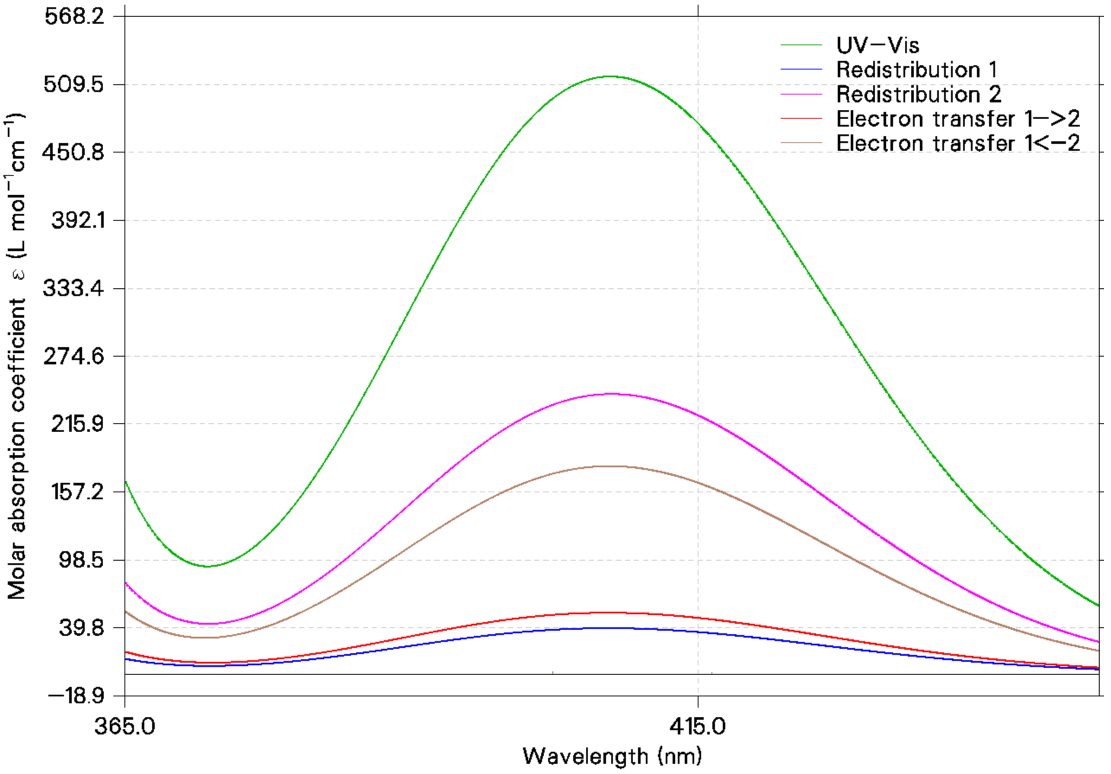

Figure S11(c). Charge transfer spectrum (CTS) of $B_6C_6N_6$ under $-F_y$ EEF of −0.015 a.u. based on Hirshfeld partition (Fragments 1 and 2 are C and B&N, respectively. The full width at half maximum (FWHM) is 0.3 eV. The absorption band range is at 365–450 nm.)

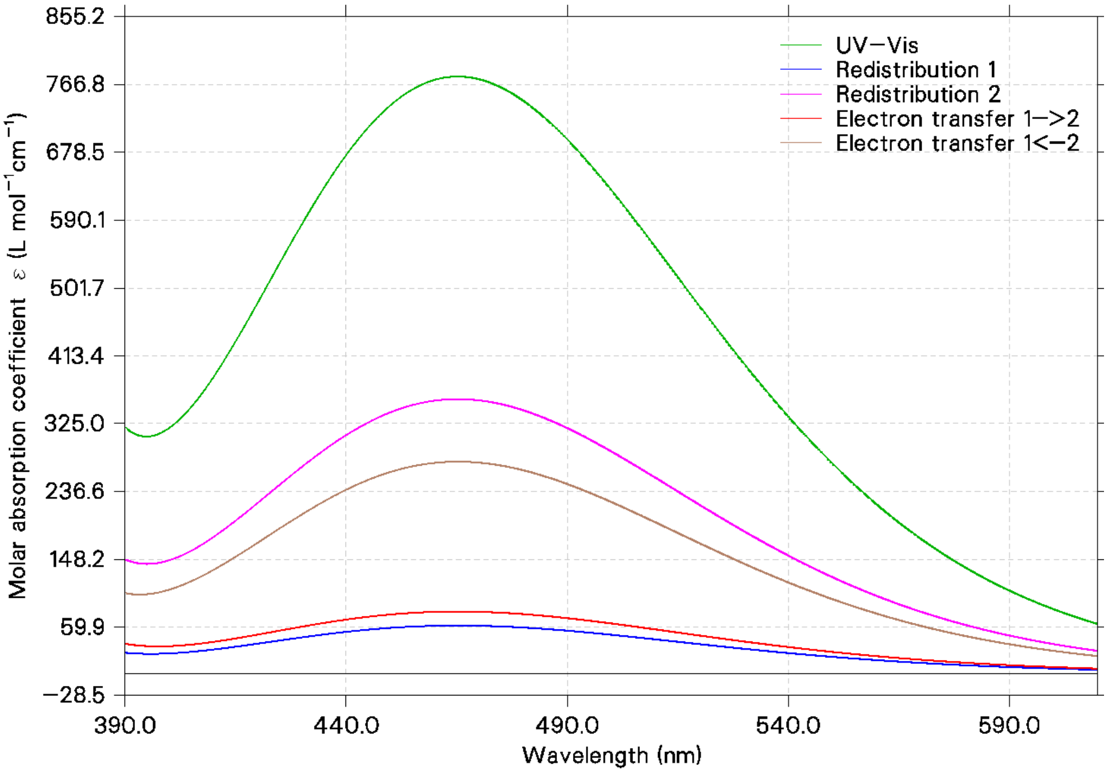

Figure S11(d). Charge transfer spectrum (CTS) of $B_6C_6N_6$ under $-F_y$ EEF of −0.02 a.u. based on Hirshfeld partition (Fragments 1 and 2 are C and B&N, respectively. The full width at half maximum (FWHM) is 0.3 eV. The absorption band range is at 390−610 nm.)

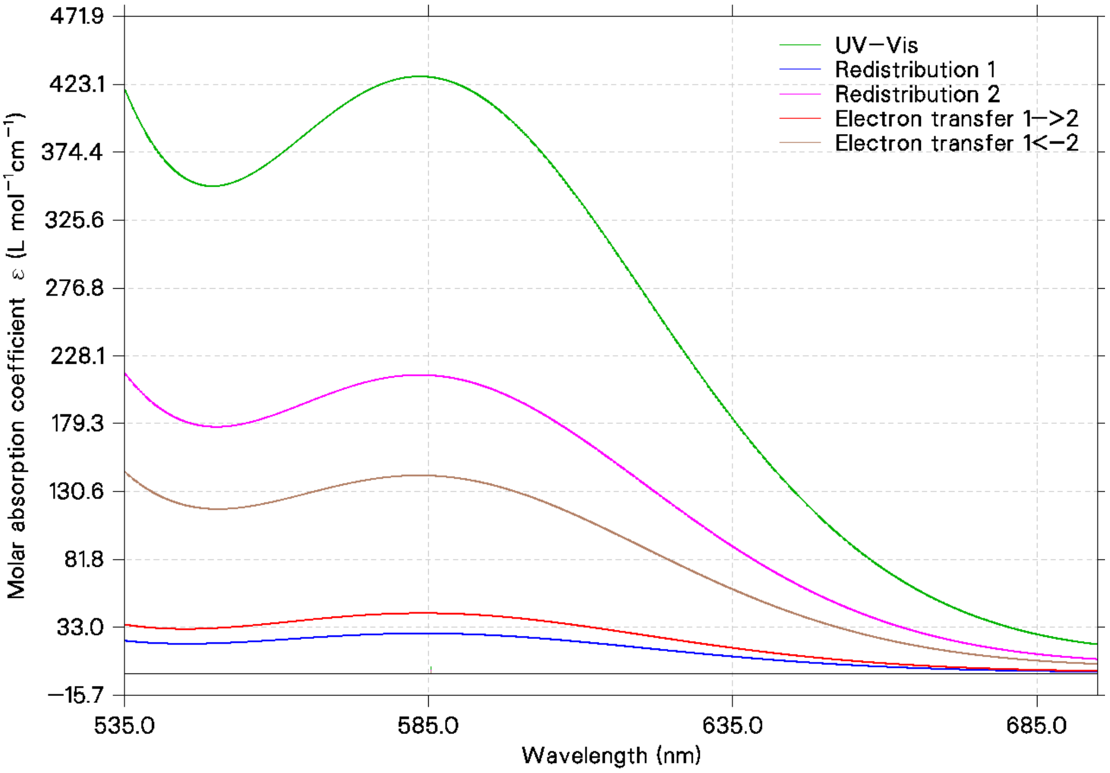

Figure S11(e). Charge transfer spectrum (CTS) of $B_6C_6N_6$ under $-F_y$ EEF of −0.025 a.u. based on Hirshfeld partition (Fragments 1 and 2 are C and B&N, respectively. The full width at half maximum (FWHM) is 0.3 eV. The absorption band range is at 535−695 nm.)

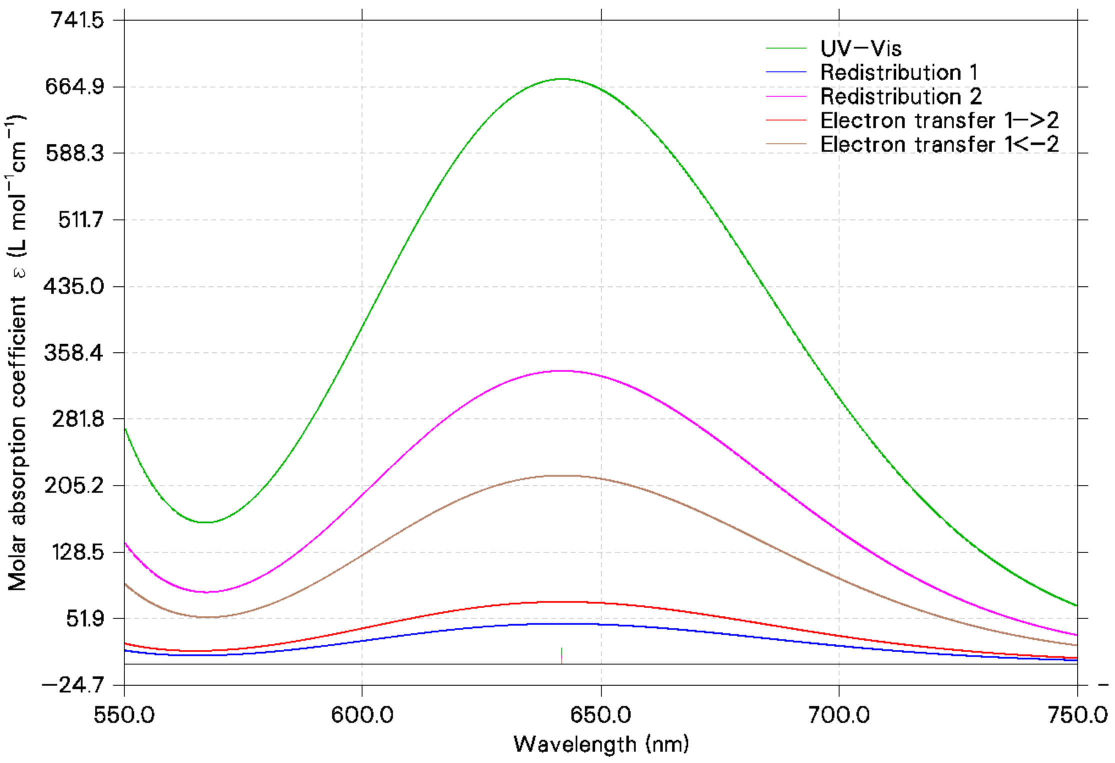

Figure S11(f). Charge transfer spectrum (CTS) of $B_6C_6N_6$ under $-F_y$ EEF of −0.0275 a.u. based on Hirshfeld partition (Fragments 1 and 2 are C and B&N, respectively. The full width at half maximum (FWHM) is 0.3 eV. The absorption band range is at 535−695 nm.)

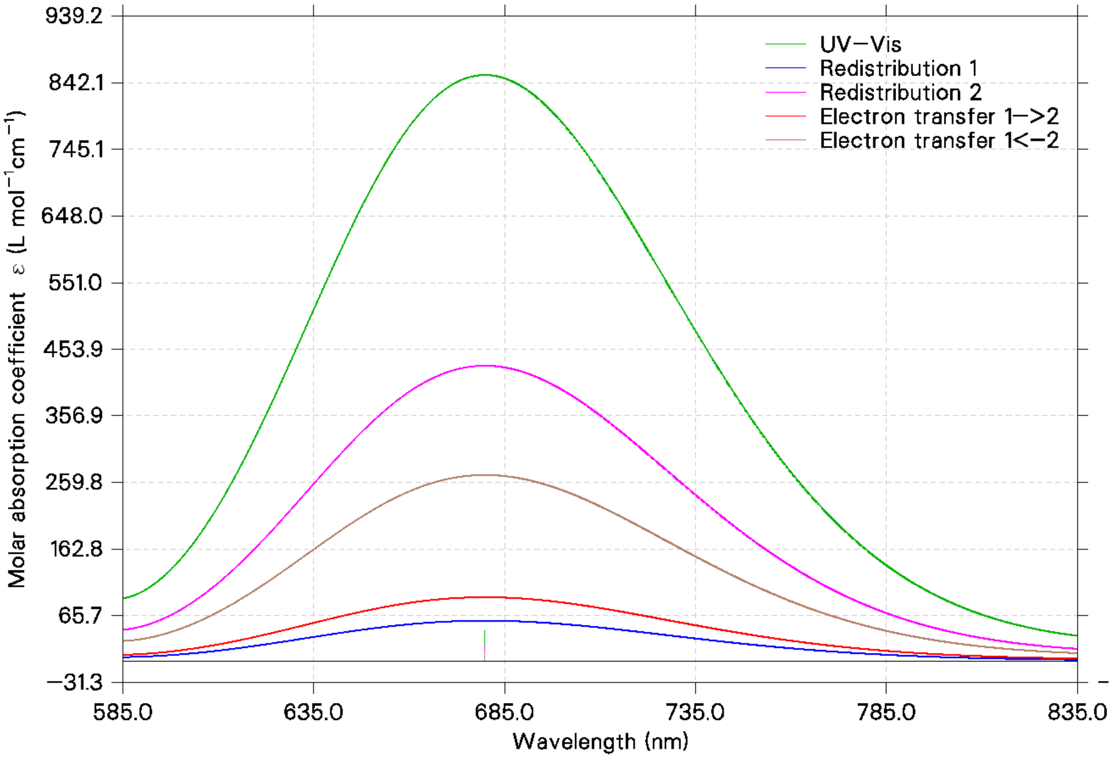

Figure S11(g). Charge transfer spectrum (CTS) of $B_6C_6N_6$ under $-F_y$ EEF of −0.029 a.u. based on Hirshfeld partition (Fragments 1 and 2 are C and B&N, respectively. The full width at half maximum (FWHM) is 0.3 eV. The absorption band range is at 585−835 nm.)

Table S3. Excited energy (in eV), excited wavelength (in nm), oscillator strength (*f*) and main molecular orbital transitions of 20 excited states of of $B_6C_6N_6$ without EEF and of $B_6C_6N_6$ under different $-F_y$ EEFs at gas phase

EEF = 0				
State	Energy/eV	λ/nm	*f*	Mayor MO transitions
1	3.5240	351.83	0.00000	H→L+1 (45.8%); H-1→L (45.8%)
**2**	3.5915	345.22	0.00000	H-3→L (24.8%); H-2→L+1 (24.8%); H-1→L+3 (22.2%); H→L+2 (22.2%)
3	3.6253	342.00	0.00000	H-3→L+3 (45.2%); H-2→L+2 (45.2%)
4	3.8377	323.07	0.00000	H-1→L (33.1%); H→L+1 (33.1%); H-4→L+1 (12.1%)
5	3.8378	323.06	0.00000	H-1→L+1 (33.1%); H→L (33.1%); H-4→L (12.1%)
6	3.9197	316.31	0.00050	H→L+3 (18.6%); H-1→L+2 (18.6%); H-2→L (18.4%); H-3→L+1 (18.4%); H-4→L+3 (6.9%); H-5→L (6.8%)
7	3.9198	316.30	0.00050	H→L+2 (18.6%); H-1→L+3 (18.6%); H-2→L+1 (18.4%); H-3→L (18.4%); H-4→L+2 (6.9%); H-5→L+1 (6.8%)
8	3.9368	314.94	0.00000	H-2→L+2 (32.6%); H-3→L+3 (32.5%); H-5→L+2 (10.9%)
9	3.9368	314.94	0.00000	H-3→L+2 (32.5%); H-2→L+3 (32.5%); H-5→L+3 (10.9%)
10	4.2464	291.97	0.00000	H→L+2 (25.8%); H-1→L+3 (25.8%); H-3→L (23.3%); H-2→L+1 (23.3%)
11	4.7978	258.42	0.00000	H-1→L+1 (24.3%); H→L (24.3%); H-3→L+2 (15.6%); H-2→L+3 (15.6%); H-4→L+5 (8.2%); H-5→L+4 (7.5%)
12	4.8002	258.29	0.00000	H-5→L+3 (29.4%); H-2→L+4 (25.7%); H-4→L (14.1%); H→L+5 (9.7%)
13	4.8002	258.29	0.00000	H-5→L+2 (29.4%); H-3→L+4 (25.7%); H-4→L+1 (14.1%); H-1→L+5 (9.7%)
14	4.8645	254.88	0.00030	H-2→L+3 (23.0%); H-3→L+2 (23.0%); H→L (17.8%); H-1→L+1 (17.8%); H-5→L+4 (9.5%); H-4→L+5 (5.1%)
15	4.8656	254.82	0.00000	H-3→L+1 (21.8%); H-2→L (21.8%); H-1→L+2 (19.4%); H→L+3 (19.4%); H-4→L+4 (7.7%); H-5→L+5 (6.6%)
16	4.9433	250.81	0.00010	H-4→L+3 (22.7%); H-5→L (22.6%); H→L+4 (20.5%); H-2→L+5 (15.5%)
17	4.9434	250.81	0.00010	H-4→L+2 (22.7%); H-5→L+1 (22.6%); H-1→L+4 (20.5%); H-3→L+5 (15.5%)
18	4.9503	250.46	0.00000	H-4→L+1 (33.6%); H-1→L+5 (22.8%); H-5→L+2 (12.9%); H-3→L+4 (11.7%)
19	4.9503	250.46	0.00000	H-4→L (33.6%); H→L+5 (22.8%); H-5→L+3 (12.9%); H-2→L+4 (11.7%)
20	5.3227	232.93	0.00000	H→L+3 (24.5%); H-1→L+2 (24.5%); H-2→L (22.9%); H-3→L+1 (22.9%)

EEF = -0.01 a.u.				
State	Energy/eV	λ/nm	*f*	Mayor MO transitions
1	3.2875	377.14	0.00000	H-1→L+2 29.1%, H→L+3 22.2%, H-2→L 13.3%, H-1→L 12.6%, H-3

				→L+1 7.7%
**2**	3.3290	372.44	0.00000	H-3→L+1 29.4%, H-1→L 22.2%, H→L+3 20.0%, H-1→L+2 10.1%, H-2→L+2 6.0%
3	3.3364	371.61	0.00000	H-2→L 37.6%, H→L+3 18.0%, H→L+1 16.6%, H-1→L+2 6.5%
4	3.3371	371.53	0.00230	H→L+2 26.8%, H-1→L+3 26.3%, H-3→L 10.7%, H-2→L+1 10.2%, H→L 9.8%, H-1→L+1 6.0%
5	3.4041	364.22	0.00000	H→L+1 26.9%, H-3→L+1 20.3%, H-2→L 13.5%, H-1→L 12.7%, H-3→L+3 11.2%, H-2→L+2 5.2%
6	3.4113	363.45	0.00100	H-2→L+1 21.6%, H-3→L 19.2%, H→L 18.8%, H-1→L+1 15.7%, H-3→L+2 8.3%, H-2→L+3 6.9%
7	3.8899	318.73	0.00000	H-5→L 21.0%, H-1→L+2 15.4%, H-4→L+1 12.0%, H→L+3 8.4%, H-3→L+1 7.6%, H→L+1 6.3%, H-1→L 5.7%, H-1→L+5 5.1%
8	3.9313	315.38	0.00370	H-1→L+3 27.1%, H-4→L 19.7%, H-3→L 18.1%, H-5→L+1 9.1%, H→L 6.2%
9	3.9469	314.13	0.00040	H-5→L 17.2%, H-4→L+1 15.6%, H→L+3 12.2%, H-1→L+2 10.9%, H-3→L+1 10.0%, H→L+1 9.1%, H→L+6 5.7%
10	4.0276	307.84	0.00450	H→L+2 29.7%, H-2→L+1 28.2%, H-3→L 12.1%, H-1→L+3 8.3%, H-5→L+1 7.9%
11	4.4571	278.17	0.00000	H-5→L 23.1%, H-1→L+5 21.8%, H-4→L+1 15.9%, H→L+6 6.7%, H-1→L+4 5.0%
12	4.5189	274.37	0.45250	H-1→L+1 46.9%, H→L 42.0%
13	4.5563	272.12	0.00000	H-4→L+1 31.3%, H→L+1 11.3%, H→L+6 10.7%, H-5→L 9.6%, H-3→L+3 9.3%, H-1→L+5 8.1%
14	4.5648	271.61	0.01900	H-4→L 21.7%, H-5→L+1 17.9%, H→L 12.8%, H→L+5 11.3%, H-1→L+6 9.6%
15	4.6867	264.54	0.00010	H-2→L+2 42.6%, H-1→L 20.4%, H-5→L+2 5.6%
16	4.7725	259.79	0.00370	H-2→L+3 27.6%, H-3→L+2 19.2%, H-1→L+1 13.2%, H-3→L+5 5.7%
17	4.7912	258.77	0.00000	H-3→L+3 39.5%, H-3→L+6 10.3%, H→L+1 10.3%, H→L+6 9.7%
18	4.9841	248.76	0.00030	H-5→L+2 35.0%, H-2→L+5 21.0%, H-2→L+4 7.2%, H-9→L 6.2%, H-4→L+3 5.4%
19	5.1661	240.00	0.02310	H-4→L+2 24.2%, H-5→L+3 21.6%, H-3→L+5 10.9%, H-2→L+6 10.8%
20	5.2172	237.65	0.00040	H-4→L+3 42.3%, H-3→L+6 18.1%, H-8→L+1 8.3%, H-5→L+2 5.6%

EEF = -0.015 a.u.				
State	Energy/eV	λ/nm	f	Mayor MO transitions
1	2.8453	435.75	0.00000	H-1→L 40.6%, H-2→L 35.3%, H-2→L+2 9.6%
**2**	2.9556	419.49	0.00000	H-1→L+2 35.8%, H-2→L 32.4%, H-1→L 13.0%
3	2.9790	416.19	0.00240	H→L 36.3%, H-1→L+1 21.2%, H→L+2 14.3%, H-1→L+3 9.7%
**4**	3.0186	410.73	0.00000	H→L+1 45.0%, H→L+3 34.1%, H-3→L+3 5.1%
5	3.0813	402.38	0.00380	H-3→L 33.0%, H-2→L+1 30.5%, H-1→L+3 12.8%, H→L+2 11.1%
6	3.1310	395.99	0.00000	H-3→L+1 60.6%, H→L+3 18.3%, H-3→L+3 6.1%, H→L+1 5.6%

7	3.6880	336.18	0.00000	H-5→L 29.3%, H-1→L+2 27.6%, H-1→L 13.0%, H-1→L+5 8.8%, H-2→L 5.6%
8	3.6953	335.52	0.02520	H-1→L+3 36.0%, H-3→L 25.7%, H→L 14.2%, H-4→L 10.2%
9	3.7702	328.85	0.00040	H→L+3 25.0%, H→L+1 22.5%, H-3→L+1 15.4%, H-4→L+1 11.9%, H→L+7 9.9%
10	3.8026	326.05	0.00410	H→L+2 29.2%, H-2→L+1 24.5%, H-1→L+1 13.1%, H-5→L+1 9.9%, H→L+5 7.7%
11	3.9553	313.46	0.33150	H-1→L+1 48.6%, H→L 37.6%
12	4.2141	294.21	0.00000	H-5→L 41.3%, H-1→L+5 16.5%, H-1→L 9.9%, H-1→L+6 6.0%
13	4.3226	286.83	0.00450	H-4→L 34.7%, H-5→L+1 22.8%, H-1→L+1 5.2%
14	4.3857	282.70	0.00000	H-4→L+1 62.9%, H-3→L+3 8.0%, H→L+1 6.5%, H→L+7 6.2%
15	4.4339	279.63	0.00000	H-2→L+2 33.8%, H-1→L 11.1%, H-1→L+5 9.7%, H-2→L+5 7.9%, H-1→L+6 6.8%
16	4.6149	268.66	0.00340	H-2→L+3 21.4%, H-3→L+2 14.0%, H-1→L+7 9.6%, H→L+5 8.6%, H-1→L+1 7.0%, H-3→L+5 6.2%, H→L+6 5.4%
17	4.7006	263.76	0.00010	H-3→L+3 35.4%, H→L+7 21.0%, H-3→L+7 12.8%, H→L+1 7.0%, H-6→L+3 5.2%
18	4.9183	252.09	0.00060	H-5→L+2 35.8%, H-2→L+5 22.7%, H-9→L 6.3%, H-2→L+6 6.0%
19	4.9669	249.62	1.02710	H→L+2 29.3%, H-3→L 20.7%, H-1→L+3 17.5%, H-2→L+1 14.1%
20	5.1408	241.18	0.00060	H-1→L+5 14.3%, H-2→L+2 10.4%, H-1→L+6 9.5%, H-1→L+2 9.0%, H-5→L+2 7.3%, H-5→L 6.0%, H-2→L 5.7%, H-2→L+5 5.1%, H-1→L+8 5.0%

EEF = -0.02 a.u.				
State	Energy/eV	λ/nm	f	Mayor MO transitions
1	2.4797	500.00	0.00000	H-1→L 55.0%, H-2→L+2 34.1%
**2**	2.4982	496.29	0.00000	H-1→L+2 55.0%, H-2→L 33.1%
3	2.6601	466.09	0.01460	H-1→L+1 34.4%, H→L+2 31.7%, H-3→L 16.7%, H-2→L+3 9.9%
**4**	2.6744	463.60	0.00440	H→L 41.4%, H-3→L+2 20.6%, H-2→L+1 15.2%, H-1→L+3 14.3%
5	2.7153	456.61	0.00020	H→L+1 71.3%, H-3→L+3 19.6%
6	2.8435	436.03	0.00000	H-3→L+1 48.2%, H→L+3 44.8%
7	3.4838	355.89	0.00190	H-2→L+1 38.5%, H→L 29.6%, H-1→L+3 19.1%, H-4→L+1 7.2%
8	3.6668	338.13	0.00010	H-4→L 38.8%, H-2→L 32.3%, H-1→L+2 22.4%
9	3.6952	335.53	0.00000	H-2→L+2 38.2%, H-4→L+2 27.4%, H-1→L 21.9%
10	3.9891	310.81	0.00410	H-3→L+2 39.5%, H-1→L+3 24.0%, H-1→L+7 8.3%, H-7→L+2 8.0%, H→L 5.3%
11	4.0062	309.48	0.12830	H→L+2 56.7%, H-3→L 29.9%, H-1→L+1 7.3%
12	4.0207	308.36	0.00000	H→L+3 33.7%, H-3→L+1 32.2%, H→L+7 17.9%, H-7→L+1 8.6%
13	4.1458	299.06	0.43550	H-1→L+1 52.1%, H-3→L 18.5%, H-2→L+3 17.5%
14	4.3711	283.65	0.00080	H-4→L 37.8%, H-2→L 19.0%, H-1→L+2 16.1%, H-2→L+5 8.0%, H-2→L+6 6.9%

15	4.3867	282.64	0.00020	H-3→L+3 62.9%, H→L+1 21.5%, H-3→L+7 5.9%
16	4.3957	282.06	0.18250	H-3→L+2 29.0%, H→L 19.2%, H-2→L+1 16.7%, H-1→L+3 14.9%, H-4→L+1 7.7%
17	4.3966	282.00	0.00000	H-4→L+2 46.0%, H-1→L+6 12.2%, H-2→L+2 8.2%, H-1→L+10 7.1%
18	4.6040	269.30	0.00470	H-7→L+2 32.2%, H-4→L+1 29.7%, H-2→L+1 6.9%, H-1→L+7 6.7%, H→L+6 5.4%
19	4.6607	266.02	0.00000	H-7→L+1 72.7%, H→L+7 9.8%
20	4.6906	264.32	0.00000	H-1→L+6 22.5%, H-1→L+5 18.6%, H-1→L 16.8%, H-2→L+2 10.5%, H-4→L+2 9.7%, H-1→L+10 5.7%

EEF = -0.025 a.u.				
State	Energy/eV	λ/nm	*f*	Mayor MO transitions
1	1.6155	767.47	0.00020	H-2→L 53.1%, H-1→L 32.4%, H-2→L+2 7.9%
2	2.1172	585.60	0.00000	H-1→L 47.5%, H-2→L 25.3%, H-1→L+3 9.0%, H-1→L+2 8.5%
3	2.1177	585.47	0.00470	H→L 69.2%, H-1→L+1 12.6%
4	2.3544	526.61	0.00000	H→L+1 66.9%, H→L+4 27.1%
5	2.5331	489.46	0.01370	H-3→L 41.3%, H-2→L+1 28.6%, H-1→L+4 12.0%
6	2.8202	439.63	0.00040	H-3→L+1 54.7%, H→L+4 27.5%, H→L+1 7.1%
7	2.8424	436.20	0.12300	H-1→L+1 45.8%, H→L 27.3%, H→L+2 5.8%, H-1→L+4 5.3%, H →L+3 5.2%
8	2.9403	421.67	0.00010	H-1→L+3 25.3%, H-1→L+2 23.6%, H-1→L+5 17.9%, H-1→L 11.4%, H-5→L 6.3%
9	2.9770	416.47	0.03250	H-3→L 34.8%, H-1→L+4 26.6%, H-2→L+1 23.4%
10	3.1140	398.15	0.02690	H-1→L+1 31.4%, H-2→L+1 17.5%, H→L+2 12.6%, H→L+3 11.9%, H→L+5 9.3%
11	3.1380	395.11	0.00010	H→L+4 34.7%, H-3→L+1 28.2%, H→L+1 19.1%
12	3.5031	353.93	0.00020	H-5→L 43.1%, H-1→L+6 13.3%, H-2→L+6 9.5%
13	3.5670	347.59	0.00010	H-5→L 18.4%, H-2→L+2 16.1%, H-1→L+6 9.7%, H-2→L+6 9.6%, H-2→L+5 7.4%, H-2→L+3 7.1%
14	3.7078	334.39	0.04820	H-4→L 54.4%, H-5→L+1 9.8%, H-1→L+4 7.2%, H→L+2 7.0%
15	4.0078	309.36	0.09900	H→L+2 23.0%, H→L+6 13.5%, H→L+5 13.4%, H-1→L+8 9.9%, H→L+7 6.5%
16	4.0390	306.97	0.00030	H-1→L+2 47.2%, H-1→L+3 29.3%
17	4.0748	304.27	0.00020	H-1→L+5 23.6%, H-2→L+6 20.8%, H-2→L+5 9.6%, H-1→L+2 7.1%, H-1→L+6 5.7%, H-1→L+7 5.4%
18	4.1242	300.63	0.05470	H→L+2 37.6%, H→L+3 16.5%, H-2→L+4 10.8%, H→L+6 6.6%
19	4.1424	299.31	0.00000	H-4→L+1 81.5%
20	4.1625	297.86	0.40080	H→L+3 40.1%, H-1→L+4 14.1%, H-2→L+4 12.8%, H-5→L+1 7.2%

EEF = -0.0275 a.u.				
State	Energy/eV	λ/nm	*f*	Mayor MO transitions

1	1.4228	871.41	0.00030	H-2→L 52.0%, H-1→L 33.8%, H-2→L+2 7.6%
2	1.9289	642.77	0.00000	H-1→L 50.9%, H-2→L 27.6%, H-1→L+3 8.4%
3	1.9316	641.87	0.00750	H→L 75.7%, H-1→L+1 11.1%
4	2.2112	560.71	0.00000	H→L+1 71.3%, H→L+4 23.8%
5	2.4884	498.25	0.01200	H-3→L 37.4%, H-2→L+1 28.7%, H-1→L+4 15.0%
6	2.6751	463.48	0.11910	H-1→L+1 50.9%, H→L 19.2%, H-2→L+1 11.8%, H→L+3 6.3%
7	2.7626	448.80	0.00020	H→L+4 37.1%, H-3→L+1 31.8%, H→L+1 10.7%
8	2.7760	446.63	0.00040	H-1→L+3 29.5%, H-1→L+5 21.0%, H-1→L+2 14.0%, H-3→L+1 7.3%, H-1→L 7.1%
9	2.8138	440.63	0.02060	H-3→L 40.4%, H-1→L+4 26.0%, H-2→L+1 16.6%, H-3→L+2 5.2%
10	2.9538	419.74	0.02770	H-1→L+1 31.5%, H-2→L+1 17.6%, H→L+3 14.8%, H→L+5 10.7%, H→L+2 8.9%
11	3.0214	410.35	0.00020	H-3→L+1 44.8%, H→L+4 25.4%, H→L+1 12.1%, H-3→L+4 5.2%
12	3.3483	370.29	0.00030	H-5→L 37.9%, H-1→L+6 13.2%, H-2→L+6 12.1%, H-1→L+7 5.5%
13	3.4045	364.18	0.00020	H-5→L 21.2%, H-2→L+2 13.4%, H-2→L+6 9.6%, H-2→L+3 9.6%, H-2→L+5 8.6%, H-1→L+6 7.5%
14	3.5385	350.39	0.05410	H-4→L 49.7%, H→L+2 12.4%, H-1→L+4 10.9%, H-5→L+1 6.3%
15	3.7387	331.62	0.00050	H-1→L+2 65.5%, H-1→L+3 12.6%
16	3.7839	327.66	0.03220	H→L+2 59.4%, H→L+5 15.2%, H-4→L 5.5%
17	3.8560	321.54	0.00020	H-1→L+5 26.7%, H-2→L+6 17.1%, H-1→L+3 11.4%, H-2→L+2 9.3%
18	3.9079	317.27	0.09220	H→L+3 39.1%, H→L+6 15.4%, H-1→L+8 7.6%, H→L+7 5.5%
19	4.0214	308.31	0.28140	H-2→L+4 24.7%, H→L+3 12.5%, H-1→L+4 8.8%, H-3→L+6 8.1%, H-5→L+1 6.8%
20	4.0665	304.89	0.00000	H-4→L+1 71.9%, H-6→L+1 6.7%

EEF = -0.029 a.u.				
State	Energy/eV	λ/nm	f	Mayor MO transitions
1	1.3265	934.67	0.00030	H-2→L 50.6%, H-1→L 35.0%, H-2→L+2 7.6%
2	1.8203	681.12	0.00000	H-1→L 52.0%, H-2→L 29.3%, H-1→L+3 6.9%
3	1.8238	679.81	0.00950	H→L 78.4%, H-1→L+1 10.7%
4	2.1289	582.39	0.00000	H→L+1 73.9%, H→L+4 21.8%
5	2.4681	502.35	0.01050	H-3→L 31.0%, H-2→L+1 27.2%, H-1→L+4 18.5%
6	2.5887	478.94	0.11490	H-1→L+1 49.4%, H-2→L+1 21.3%, H→L 14.5%, H→L+3 5.0%
7	2.6852	461.73	0.00010	H-1→L+3 32.5%, H-1→L+5 27.2%, H-1→L+2 13.2%, H-1→L 6.2%
8	2.7246	455.05	0.00050	H→L+4 49.0%, H-3→L+1 26.8%, H→L+1 12.0%
9	2.7428	452.04	0.01860	H-3→L 46.3%, H-1→L+4 22.4%, H-2→L+1 9.6%, H-3→L+2 6.5%
10	2.8665	432.53	0.02950	H-1→L+1 31.9%, H-2→L+1 17.4%, H→L+3 14.5%, H→L+5 11.5%, H→L+2 8.5%
11	2.9752	416.73	0.00020	H-3→L+1 57.1%, H→L+4 17.5%, H→L+1 7.8%, H-3→L+4 6.2%
12	3.2617	380.12	0.00020	H-5→L 41.6%, H-2→L+6 10.3%, H-1→L+6 10.0%, H-2→L+2 7.8%

13	3.3178	373.69	0.00040	H-5→L 14.7%, H-2→L+6 12.8%, H-2→L+3 11.2%, H-2→L+5 11.0%, H-2→L+2 10.7%, H-1→L+6 8.9%, H-1→L+7 6.3%
14	3.4344	361.01	0.05350	H-4→L 43.6%, H→L+2 21.5%, H-1→L+4 11.8%
15	3.5666	347.63	0.00070	H-1→L+2 68.0%, H-1→L+3 8.2%, H-1→L+5 5.7%
16	3.6250	342.03	0.00730	H→L+2 55.3%, H→L+5 13.8%, H-4→L 10.7%, H→L+3 5.4%
17	3.7267	332.69	0.00020	H-1→L+5 27.9%, H-1→L+3 16.7%, H-2→L+2 13.5%, H-2→L+6 13.1%
18	3.8022	326.09	0.12140	H→L+3 45.4%, H→L+6 12.1%, H→L+5 8.0%, H-1→L+8 5.5%, H-1→L+4 5.1%
19	3.9569	313.34	0.18420	H-2→L+4 25.1%, H→L+6 8.9%, H-3→L+6 8.0%, H→L+3 7.3%, H→L+7 6.3%, H-1→L+8 6.1%, H-1→L+4 5.5%
20	3.9673	312.52	0.00050	H-2→L+2 30.2%, H-1→L+6 14.4%, H-5→L 10.7%, H-2→L+7 7.2%, H-2→L+5 6.9%, H-2→L 5.1%

EEF	state	hole and electron	C_{ele} and C_{hole}	Sr
-0.01	S4	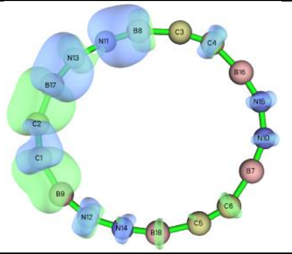	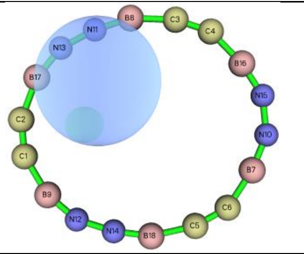	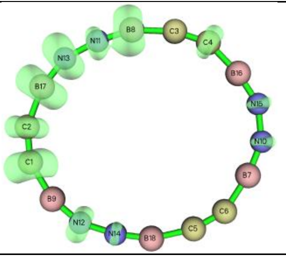
-0.015	S3	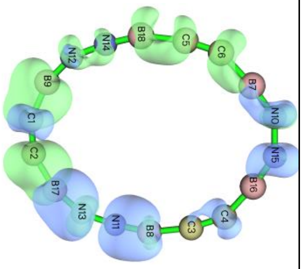	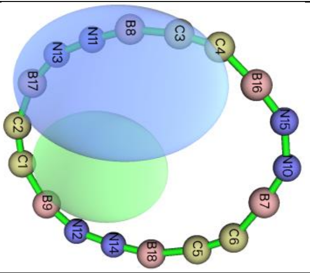	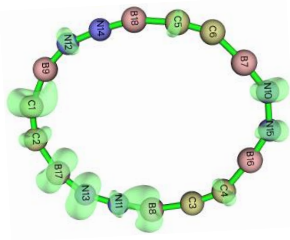
-0.02	S3	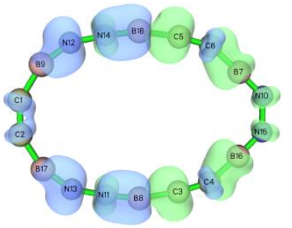	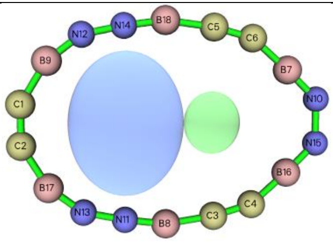	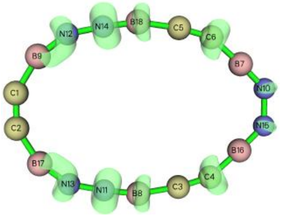
-0.025	S3	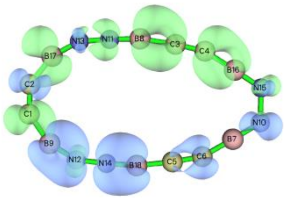	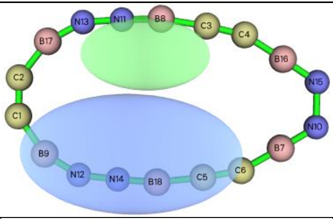	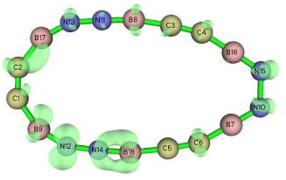
-0.0275	S3	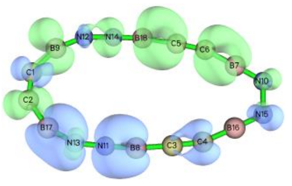	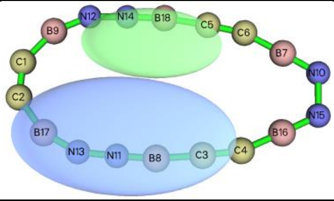	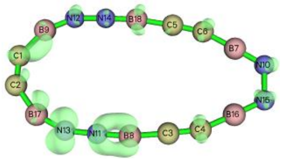

-0.029	S3	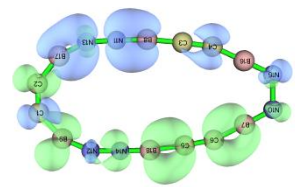	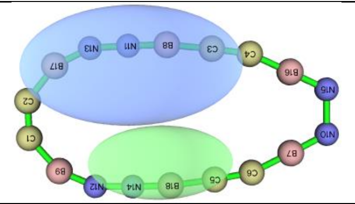	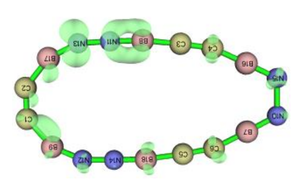

Figure S12. Excited-state hole-electron analyses of $B_6C_6N_6$ under various $-F_y$ EEF. For the isosurface of hole and electron distribution and the isosurface of C_{ele} and C_{hole} functions, green and blue stand for electron and hole distributions, respectively. Isovalue = 0.002 a.u. except for the isosurface of C_{ele} and C_{hole} functions of −0.015 a.u. Isovalue = 0.0015 a.u. is adopted for the isosurface of C_{ele} and C_{hole} functions of −0.015 a.u., because its C_{ele} functions cannot displays at isovalue = 0.002 a.u.

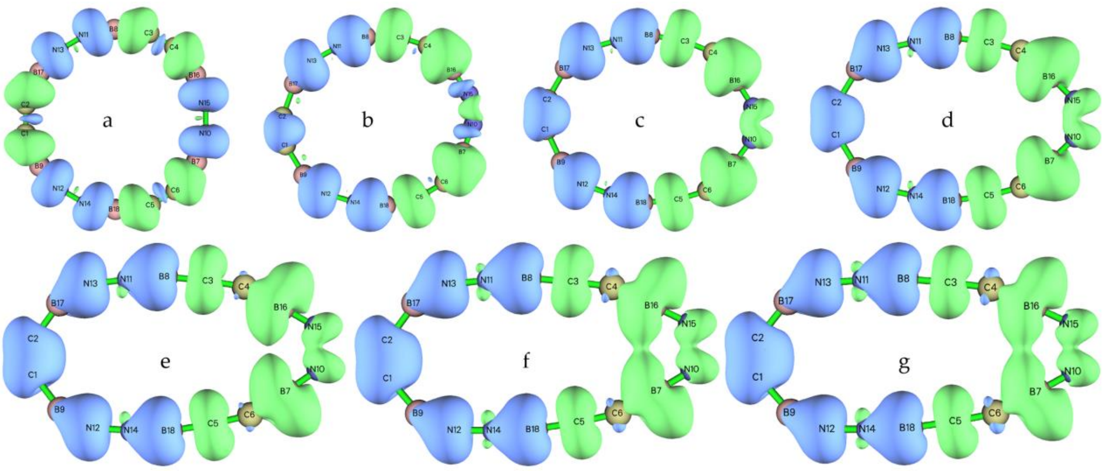

Figure S13. Isosurface of orbital-weighted dual descriptor of $B_6C_6N_6$ without EEF and of $B_6C_6N_6$ under different $-F_y$ EEFs (a) 0, (b) −0.01, (c) −0.015, (d) −0.02, (e) −0.025, (f) −0.0275, and (g) −0.029 a.u. Blue isosurfaces denote negative orbital-weighted dual descriptor values, and green isosurfaces denote positive values. (Isovalue = 0.001 a.u.)
